# From crisis to new routine: shifts in urban shopping mobility and socioeconomic inequality during and after the COVID-19 shock

**DOI:** 10.3389/fdata.2026.1776172

**Published:** 2026-09-15

**Authors:** Yilun Xu, Mohsen Bahrami, Alex Pentland

**Affiliations:** 1 Laboratory for Innovation Science at Harvard, Harvard University, Allston, MA, United States; 2 MIT Initiative on the Digital Economy, Sloan School of Management, Massachusetts Institute of Technology, Cambridge, MA, United States; 3 MIT Connection Science, Institute for Data, Systems, and Society, Massachusetts Institute of Technology, Cambridge, MA, United States

**Keywords:** COVID-19 pandemic, heterogeneous mobility shifts, retail accessibility, socioeconomic inequality, urban shopping mobility

## Abstract

**Introduction:**

This study examines the impact of the COVID-19 pandemic on revealed shopping-location patterns and their persistence during the early recovery period. It focuses on urban shopping mobility in New York City and investigates whether observed store-selection patterns returned to, or remained different from, their pre-pandemic baseline by 2021 across different socioeconomic communities.

**Methods:**

We use large-scale mobility and place datasets together with census information to analyze visits to general merchandise and department stores in New York City. A modified Huff gravity model and a metaheuristic calibration method are used to quantify temporal shifts in store-selection patterns. We also use unsupervised learning techniques and statistical inference models to examine heterogeneity across socioeconomic communities and evaluate changes before, during, and after the COVID-19 shock.

**Results:**

The proposed model captures the dynamics of shopping-location decisions and temporal visit patterns under changing pandemic-era constraints. The results suggest that New Yorkers' revealed store-selection patterns changed substantially during the COVID-19 shock, with increased emphasis on store area, chain loyalty, and nearby points of interest, and reduced sensitivity to customer-store distance. These patterns did not fully revert to their 2019 baseline by 2021, although 2021 is interpreted as an early recovery and reopening period rather than a fully post-pandemic equilibrium.

**Discussion:**

The findings highlight the societal dimension of urban shopping mobility disruptions during crises and show that mobility-based store-selection patterns vary across socioeconomic communities. The results can help urban planners, managers, and marketers better understand heterogeneous shifts in shopping mobility and adapt strategies under crisis and recovery conditions.

## 1 Introduction

The COVID-19 pandemic, as a worldwide public health emergency, has forced individuals to alter their daily routines, particularly those residing in urban areas (Bavel et al., 2020). Mandates such as social distancing and government interventions aimed at reducing the transmission of the disease, such as shelter-in-place orders and the closure of non-essential businesses, coupled with the fear of contracting the virus and its uncertain consequences, prompted individuals to implement self-imposed precautions, disrupting their regular activities (Cronin and Evans, 2020; Zion et al., 2022). The impact of these measures has been particularly significant in densely populated cities such as New York City (NYC), which implemented strict city-wide lockdowns referred to as the “micro-cluster” strategy (Birge et al., 2022).

Much recent research has illuminated the dynamics of such behavioral changes in various contexts including human mobility (Lee et al., 2020; Chan et al., 2020; Cronin and Evans, 2020; Chang et al., 2021), physical activity (e.g., walking behavior) (Dwyer et al., 2020; Hunter et al., 2021; Stockwell et al., 2021), spending patterns (Chetty et al., 2020; Mulay et al., 2021; Liu et al., 2021; Yang et al., 2021; Chawla et al., 2022; Rose et al., 2023), shopping and consumption (Grashuis et al., 2020; Liu et al., 2021; Moon et al., 2021; Lucchini et al., 2021; Park et al., 2022), the tendency to work from home (Phillips, 2020; Klerk et al., 2021; Jones et al., 2021), and the adoption of online tools and new technologies (Lowenthal et al., 2020). More importantly, many of those studies have highlighted that the pandemic and resulting crisis have disproportionately impacted the lives of different socioeconomic groups, leading to heterogeneous behavioral shifts among them (Almagro et al., 2023; Holtz et al., 2020; Chang et al., 2021; Liu et al., 2021; Heroy et al., 2021; Maleki et al., 2022; Gür, 2022).

Certain behavioral changes have been documented as interim, temporary, and short-term in nature, resembling responses to exogenous shocks such as the impact of stimulus checks on shopping patterns (Chetty et al., 2020; Mulay et al., 2021). On the flip side, some behavioral changes are likely to be enduring and lead to the rise of a new normal even after re-openings and recovery from the economic downturn (Salon et al., 2021; Oblander and McCarthy, 2022). Corresponding persisting behavioral shifts could result from enforced acceleration of widespread digital technology adoption (e.g., online shopping, telemedicine, remote working, and virtual meetings) or the emergence of new habits due to pandemic-induced experiences and talent discovery (e.g., cooking and exercising at home) (Sheth, 2020; Kohli et al., 2020).

While the pandemic posed significant challenges for retailers, it also served as a catalyst for innovation and adaptation, particularly within the department store, general merchandise, and super-center sectors. These retailers, renowned for their diverse product offerings and immersive shopping experiences, faced substantial turmoil arising from temporary closures and decreased foot traffic, as well as surges in demand that created operational complexities and unprecedented pressure to ensure product availability and maintain a safe shopping environment. Consequently, retailers swiftly embraced omnichannel strategies and enacted digital transformations in response to the rapid shift in consumer demand toward online channels and e-commerce (Khaliq, 2021). They accelerated their investments in e-commerce platforms, improved their online shopping capabilities, and implemented contactless delivery options. This allowed customers to continue shopping while adhering to safety guidelines, underscoring that the ability to innovate and adapt has become critical for survival and long-term success of retailers in a post-pandemic retail landscape (Pantano et al., 2020; Boz et al., 2024).

Although technology can cater to both hedonic and utilitarian needs, certain consumer needs, such as social needs including personal and psychological factors, remain unfulfilled (Sheth, 2020). For instance, in today's world, retail centers serve not only as commercial establishments but also as vibrant social spaces that facilitate interactions among people from diverse socioeconomic and demographic backgrounds (Erkip, 2003; Kunc et al., 2022). Moreover, certain services are inherently physical and cannot be adequately provided through virtual channels, which underscores the enduring importance of physical retail spaces despite the increased share of online sales even after the reopening of the economy (Foroohar, 2020; Beckers et al., 2021).

Cruz-Cárdenas et al. (2021) conducted a comprehensive analysis of 70 studies examining consumer behavior and the impact of COVID-19. Their research emphasizes the significance of employing quantitative methods to estimate the extent of changes in consumer behavior from various angles. Similarly, Salon et al. (2021) collected responses through a questionnaire to assess consumer behavior before, during, and after the pandemic across different economic sectors. Their findings suggest that the changes in consumer behavior triggered by the pandemic have endured throughout the entire United States, emphasizing the significance of employing quantitative methods when preparing for a “new normal” (Salon et al., 2021).

In order to comprehensively analyze and measure the dynamics of shopping location decisions, our paper introduces a new data-driven approach, built upon the well-established Huff gravity model (Huff, 1964). By employing an enhanced multiplicative competitive interaction version of the Huff gravity model, we aim to understand and quantify the evolving patterns of urban mobility in relation to shopping location choices, taking into account the impact of contextual factors such as the COVID-19 pandemic. This approach enables us to capture and measure the complex interplay between store characteristics, customer-store proximity, neighborhood-level socioeconomic attributes, and pandemic-era constraints, providing valuable insights into the dynamics of urban shopping mobility.

Based on the existing body of literature and available data, we undertake a comprehensive analysis of revealed shopping-location patterns by considering six distinct factors. These factors include the distance between customers and stores, the floor area of the stores, the demographic similarity between customers and the residents of the store's neighborhood, customer loyalty to specific chains, and the count and diversity of amenities in the vicinity of the stores. Our objective is to address the following research questions: (1) Did significant shifts occur in revealed shopping-location patterns between the years 2018 and 2021? (2) Are there variations in the responses of different socioeconomic and demographic groups within this context? (3) Did these revealed patterns return to their pre-pandemic baseline during the early recovery period, or did they remain different by 2021? (4) What is the relative effect of the pandemic period on the mobility-based store-selection weights for each studied factor, as well as for different customer groups? Answering these questions will contribute to a better understanding of urban shopping mobility under crisis conditions, aiding businesses and policymakers in adapting strategies and making informed decisions in a rapidly evolving market landscape.

Our study contributes to the existing literature by presenting a new approach that utilizes an improved and modified version of the Huff gravity model that enables us to quantify the changes in revealed shopping-location patterns, while simultaneously considering multiple attraction factors. We acknowledge that real-world decision-making problems are inherently complex, and relying solely on unidimensional descriptive analytics may lead to oversimplification, limiting our understanding of the dynamics of customer decision-making processes. In contrast, our proposed approach offers a comprehensive evaluation model that can effectively aggregate various factors, providing a more robust and nuanced understanding of these dynamics.

Moreover, our method has the potential to contribute valuable substantive insights into the factors shaping urban shopping mobility and customer shopping location decisions before, during, and after the COVID-19 shock through quantitative analysis. By utilizing our model, we are able to effectively capture the dynamics of shopping location choices and identify changes in visit patterns. These findings can provide managers and marketers with a deeper understanding of evolving store-selection patterns under changing mobility, health-risk, and operational constraints, enabling them to adapt and enhance their strategies to remain competitive. Additionally, the validation of our model is demonstrated by the stable parameter values obtained during the pre-pandemic years, indicating the consistency of revealed shopping-location patterns in the absence of exogenous shocks.

The remainder of this paper is structured as follows. In Section 2, we conduct a comprehensive review of existing literature on the impact of the pandemic on consumer behavior. Section 3 provides a detailed background of the model we used in this study. In Section 4, we outline our proposed methodology, describe the datasets utilized, and explain the analytical framework. We then proceed to investigate the case of retail business, focusing specifically on general merchandise and department stores in New York City (NYC), and present the empirical results and their implications in Section 5. We analyze the decision-making aspects of selecting a physical store for shopping purposes that gained more importance during the COVID-19 pandemic as individuals sought to balance their needs with the risk of infection and changing mobility constraints. Additionally, we explore the heterogeneity in changes to store selection criteria among different socioeconomic communities, aiming to comprehend whether these shifts reverted to the pre-pandemic baseline by the early recovery period in 2021. Subsequently, we validate the model results and examine the robustness of our approach. Finally, we summarize the key findings in Section 6, followed by a thorough discussion of the limitations and implications of this research.

## 2 Related work

Previous studies have validated the impacts of various factors associated with the pandemic on customer behavior, both tangible and intangible (Eger et al., 2021). It has been observed that different consumer groups respond differently to consumption patterns during crisis events, often based on their individual value judgments (Eger et al., 2021). While online platforms and non-contact services have experienced significant growth during the pandemic (Chenarides et al., 2021; Nanda et al., 2021), physical stores are still considered valuable due to their ability to provide an immersive shopping experience (Puccinelli et al., 2009; Sayyida et al., 2021). Building on earlier studies that primarily focused on static comparisons at an aggregated level, our research aims to simulate the decision-making process of consumers faced with multiple store options.

Undoubtedly, consumers have embraced new behavioral patterns in light of the severe circumstances and economic upheavals triggered by the COVID-19 pandemic, backed by studies dating back as early as 2020. For example, Hall et al. (2020) spotted consumption behavior changes by region caused by the pandemic, where residents picked up a stronger preference for storage and hesitated to purchase hotel services. In particular, these behaviors during the pandemic were shaped by two pivotal factors: strict preventive measures and self-protective mindsets marked by perceived risks encompassing both health and economic concerns (Cronin and Evans, 2020; Eger et al., 2021; Wang et al., 2022).

Various customer groups exhibited different behavioral changes. Some began frequenting larger stores due to their ability to facilitate social distancing, while, others conversely shifted their shopping preferences away from larger retailers, opting instead for smaller stores, local retail centers, and out-of-town shopping alternatives (Sundström et al., 2021; Lashgari and Shahab, 2022; Ballantyne et al., 2022). Eger et al. (2021) observed that customers from various generations are likely to exhibit distinct changes in their consumption patterns when confronted with significant societal events. This observation stems from the understanding that consumers' personal experiences and values, including their shopping motivations, play a role in shaping their consumption habits (Koch et al., 2020). This insight motivated us to delve deeper into the analysis of heterogeneity in behavioral shifts among different consumer groups.

Simultaneously, there was a substantial increase in customers' preference for delivery and pick-up services (Chenarides et al., 2021; Jiang et al., 2023). However, the most notable transformation in shopping behavior occurred through a widespread transition to e-commerce platforms, particularly during periods of lockdown restrictions. This shift significantly accelerated the already thriving growth of the e-retail sector (Nanda et al., 2021; Rose et al., 2023). Several empirical studies have been conducted to demonstrate the growth of e-commerce in the retail and grocery sectors during the pandemic (Aryani et al., 2021; Naseri et al., 2021; Gomes and Lopes, 2022). It is widely acknowledged that the pandemic served as a catalyst for the advancement of online shopping platforms (Alfonso et al., 2021; Szasz et al., 2022). Furthermore, numerous studies have concentrated on comparing or examining the transition between online platforms and traditional shopping activities, with a particular emphasis on various regions worldwide (Gungordu Belbag, 2022; Timotius and Octavius, 2021; Aditantri et al., 2021; Gomes and Lopes, 2022; Rose et al., 2023; Wieland, 2021, 2023a,b).

As the pandemic promoted the prosperity of online shopping, people's tendency to visit physical stores, potentially driven by their desire to interact with products, was reaffirmed (Sayyida et al., 2021). Consequently, consumers found themselves faced with the task of choosing which stores to visit when they intended to shop under social distancing measures. Alhaimer (2022) highlighted that people's concerns during the pandemic were distinct from those in regular times, placing more emphasis on risk-related factors rather than the quality of products and services (Alhaimer, 2022). This study served as inspiration for our investigation into the impact of non-product-related factors on consumers' decisions to patronize brick-and-mortar retail stores during the pandemic. As a result, we selected general merchandise and department stores as an illustrative example. Furthermore, the presence of similar product offerings assists in minimizing the influence of the actual products on consumer behavior.

Although numerous studies have examined changes in customer behavior regarding the selection of physical retail store locations, the existing literature predominantly focuses on macro-level analysis. Related studies primarily utilize unidimensional analytics to explore collective behavioral patterns. Such studies employ longitudinal analysis to provide valuable insights into the changes in customer visit and spending patterns across different industries and geographic locations. Through diverse perspectives, these studies highlight several key findings, for example, the decline in in-store foot traffic, and increased customer in-store dwelling times (Chetty et al., 2020; Dou et al., 2020; Boyle et al., 2022; Aversa et al., 2022). According to Rossetti et al. (2022), customers tended to prioritize shopping locations with more effective social distancing regulations, even if it meant longer wait times. This highlights a decreasing emphasis on distance as a factor in consumers' decision-making process when selecting a store. Additionally, other research has highlighted the significance of store uniqueness and shopping costs as influential factors in customer decision-making when choosing a shopping location, particularly in certain industries during the pandemic (Wieland, 2023b).

While previous research emphasizes the importance of using quantitative methods to measure and understand the impact of the pandemic on consumer behavior, there are few studies that specifically examine customer decision-making and resulting shopping mobility when faced with multiple store options. To the best of our knowledge, this is also true about studies that have simulated the decision process from a computational standpoint, bringing multiple factors into account simultaneously, particularly when consumers have to choose among physical stores for shopping. Although some studies have employed psychological methodologies (Yuen et al., 2020; Naeem, 2021), there is a need for new approaches to quantify changes in revealed shopping-location patterns when customers select physical stores for shopping. In light of this, our research proposes a new method for measuring temporal shifts in observed store-selection patterns.

## 3 Theoretical background

Spatial marketing has a rich history, with its initial application dating back to the late 1920s and early 1930s, and it remains relevant today. Within spatial marketing, three fundamental concepts hold particular significance: attraction, gravitation, and spatial interaction (Cliquet, 2021). The combination of these concepts gives rise to two prevalent model types: deterministic models (Converse, 1949; Reilly, 1931) and probabilistic models (Huff, 1963). Probabilistic models offer a more flexible approach to understanding the impact of retail attraction, examining the likelihood of a customer choosing one store over another. Among these models, the original Huff gravity model is widely renowned for its simplicity and broad applicability, cementing its enduring influence in the field (Huff, 1964).

### 3.1 Huff gravity model

The Huff gravity model (Huff, 1964) is arguably the most famous probabilistic model in the spatial marketing domain. This model and its variants have been widely used and applied in many empirical studies for estimating market share in a competitive environment, mainly with the aim of determining optimal locations for new facilities or assessing a retail store's performance (Huff, 2003; Drezner and Drezner, 2004; Kim et al., 2011; De Beule et al., 2014). The original model proposed by Huff (1964) estimates the probability of customer *i* choosing store *j* or another based on the utility value that is proportional to the store's attractiveness (*A*_*j*_) and inversely proportional to customer-store distance (*D*_*ij*_). Equation 1 formally defines the utility function. The parameters α and β are used to adjust the sensitivity of the model to the two factors. Equation 2 formalizes the probability that a customer *i* visits the store *j*, where *J* denotes the set of stores that a customer *i* could potentially visit.


$$\begin{array}{l} \begin{array}{cc} U_{ij} & =\frac{A_{j}^{\alpha }}{D_{ij}^{\beta }} \end{array} \end{array}$$ (1)

$$P_{ij}=\frac{U_{ij}}{\sum_{j^{\prime }\in J}U_{ij^{\prime }}}$$ (2)

The original Huff model primarily relies on the floor area of a facility to determine its attractiveness, while the Euclidean distance between a customer base and a store location serves as an approximation of customer-store proximity. However, numerous alternative factors have been suggested in the literature to gauge facility attractiveness from various perspectives. These factors encompass diverse aspects, such as store characteristics like product pricing and parking availability (Bozkaya et al., 2010), demographic and purchase information including average customer income and brand loyalty (Seenivasan et al., 2016), and more recently, neighborhood characteristics such as the abundance and variety of amenities surrounding a store (Bahrami et al., 2022). These factors have been proposed as viable indicators of attractiveness for the respective stores, expanding the scope beyond the original measures utilized in the Huff model.

The consideration of attractions in the surrounding area of a store is founded on the principle that customers exhibit a preference for multipurpose trips over single-purpose trips (Gripsrud and Horverak, 1986; Arentze and Timmermans, 2001; Netto et al., 2023). Consequently, the quantity and variety of amenities near a store serve as an indicator of the various options available for customers to engage in multipurpose activities. The presence of a diverse range of amenities facilitates different combinations of choices for customers, enhancing the appeal of the store. Additionally, the distribution of business categories in a region can significantly influence customer behavior, and an imbalanced distribution may impact patronage patterns (France and Ghose, 2019). By incorporating these factors, a more comprehensive understanding of transient customer flows can be achieved, enabling a more accurate representation of the attractiveness of the store's location.

One major extension of the Huff model was proposed by Nakanishi and Cooper (1974). They proposed a multiplicative competitive interaction (MCI) model that replaces the store area with a product of multiple attractiveness factors, assigning tuning parameters for each factor in the product separately. Equation 3 formalizes the MCI model, where *H* is the set of store attractiveness factors *h*.


$$P_{ij}=\frac{\frac{\prod_{h\in H}A_{hj}^{\alpha_{h}}}{D_{ij}^{\beta }}}{\sum_{j^{\prime }\in J}\frac{\prod_{h\in H}A_{hj^{\prime }}^{\alpha_{h}}}{D_{ij^{\prime }}^{\beta }}}$$ (3)

Numerous extensions and adaptations of the Huff gravity model have been put forth, employing diverse functions to capture the interaction of attractiveness measures, customer-store distance proxies (e.g., Haversine distance, road network distance, travel time, and mode), and distance decay functions (e.g., exponential, weighted dimensions) across various contexts (Singleton et al., 2012; France and Ghose, 2019; Simini et al., 2021). Nevertheless, in our study, we adhere to the MCI model originally proposed by Nakanishi and Cooper (1974).

### 3.2 Calibrating model parameters

The final step in preparing the model for application involves fitting or calibrating the parameters. Several techniques have been proposed and employed for parameter tuning, including Ordinary Least Squares (OLS) (Nakanishi and Cooper, 1982; Huff and McCALLUM, 2008), Generalized Linear Model (GLM) (Beiró et al., 2018), Geographically Weighted Regression (GWR) (Suárez-Vega et al., 2015), and the more recent Particle Swarm Optimization (PSO) (Suhara et al., 2021).

The OLS approach aims to transform the non-linear model into a linear one by applying logarithmic transformation. The parameters are then optimized using OLS regression. However, this approach has limitations. It cannot handle zero observations, is sensitive to outliers, and may yield biased coefficients in the presence of heteroskedasticity.

GWR aims to account for spatial non-stationarity in the model parameters. While local models can sometimes provide a better fit compared to global models, the focus on location in the GWR approach can lead to underrating other customer preferences, particularly in densely populated areas like NYC, where stores are located in close proximity to each other and consumers' homes. Additionally, GWR may produce less accurate and highly biased results when there are insufficient local observations.

PSO is a continuous nonlinear optimization technique inspired by the foraging behavior of bird flocks (Kennedy and Eberhart, 1995) and the swarming theory, which suggests that a flock of birds searching for food can benefit from the experiences of others. In PSO, a group of particles is randomly generated within a search space to explore the optimal position. PSO does not require derivative information for optimization, and it imposes few or no assumptions about the problem, making it suitable for finding solutions in a vast space of candidate solutions (Liang et al., 2020; Freitas et al., 2020). These characteristics make PSO a problem-independent algorithm that only requires knowledge of the solution search space boundaries and the fitness evaluation metric to compare candidate solutions. As a result, it is commonly employed for model selection in machine learning (Escalante et al., 2009) and more recently for parameter calibration in the Huff model (Suhara et al., 2021). The selection of an appropriate method for parameter calibration plays a crucial role in enhancing the accuracy and performance of the model, ensuring that the results accurately reflect reality. Based on these considerations, our study utilizes a modified version of the MCI model with PSO for parameter calibration, as described in the subsequent section.

## 4 Empirical strategy and data

In this section, we first introduce our proposed modified model, which builds on the Huff gravity model, for quantifying temporal shifts in revealed shopping-location patterns. We then describe the datasets utilized in this study and finally detail the analytical setting.

### 4.1 Model modifications

The primary aim of this research is to examine the changing patterns of urban shopping mobility when residents select shopping destinations, comparing the pre-pandemic, pandemic, and early recovery periods. While the Huff gravity model and its variations were originally developed from a retailer's standpoint for trade area analysis and optimizing facility locations, we have adapted this model to measure changes in revealed shopping-location patterns when customers choose a store location for shopping. By employing this modified version, we can quantify temporal shifts in mobility-based store-selection weights and specifically evaluate the impact of the COVID-19 pandemic as a significant intervention in this context.

Since the MCI model allows taking multiple attractiveness measures into account simultaneously, we use it as the base model in this study. The first modification we make is normalizing all attractiveness factors into an interval of [1, Φ] using min-max normalization. This operation will map the minimum value of each attractiveness factor to 1, its maximum to Φ, and all other values are proportionally transformed into values within the normalization interval. Φ is tuned to optimize the model fit and can vary according to the distribution of attractiveness factors in different study cases. Details of the procedure for defining the optimal value for Φ are described in Section 4.3.4.

There are several reasons for implementing this normalization approach:

1. Eliminating the problem of 0 observations: in a multiplicative model, having a 0 value for any attractiveness factor can result in a numerator of 0, regardless of the magnitudes of other factors. For instance, if there are no other amenities and attractions near a particular store, a zero value can render the total attractiveness of the store as zero. Normalization addresses this issue by avoiding the presence of zeros in the calculation.
2. Enhancing optimization process speed and accuracy: by mapping all factors to the same interval, this approach improves the efficiency of the optimization process and provides better accuracy. It reduces the complexity of the objective function, especially when the existing factors span a wide range including values smaller and larger than 1. This modification preserves the increasing effect of the variable concerning an increase in the parameter value in the exponent.
3. Ensuring comparability and interpretability of parameters: the normalization approach facilitates comparability and interpretation of the parameters, which represent the exponents of the attractiveness factors. By having a common base range, the parameters become easier to compare and interpret, even when the attractiveness factors have significantly different ranges. For example, if the store area is measured in square meters or footage with a large magnitude and chain loyalty is defined as a fractional number within the [0,1] interval, the parameters might become difficult or impossible to interpret without normalization. With this normalization approach, the parameters can be compared across attractiveness factors and across time periods more consistently.

In the subsequent discussion, we will refer to the model parameters as revealed store-selection weights. These calibrated parameters should be interpreted as mobility-based weights inferred from observed CBG-to-store visit flows, rather than as direct measures of pure consumer preferences. In this empirical setting, the observed visit flows can be shaped by multiple demand-side and supply-side conditions, including customer needs, store characteristics, perceived health risks, mobility restrictions, store availability, operating conditions, and neighborhood-level recovery dynamics. Therefore, changes in these parameters reflect changes in revealed shopping-location patterns under changing constraints.

Utilizing this modified model, we can effectively measure the change in the relative weight associated with each attractiveness factor by comparing the magnitude of the corresponding parameter across different time periods, while keeping the other attractiveness factors constant. However, it is crucial to note that interpreting the numerical values of these parameter shifts independently may lead to misleading conclusions, as it disregards the significance of other attractiveness factors in the customer decision-making process. It is important to consider the collective impact of all attractiveness factors in order to fully understand the dynamics of revealed shopping-location patterns.

### 4.2 Data

This study incorporates three distinct large-scale datasets at a granular level: mobility patterns, places, and census datasets.

The mobility patterns dataset is derived from the geolocation data of smartphones used by individuals who have voluntarily opted into specific applications. To safeguard user privacy, the data is anonymized and aggregated based on the users' home census block groups (CBGs) on a weekly basis. CBGs are statistical areas defined by the United States Census Bureau and typically encompass a population ranging from 600 to 3,000 individuals. These CBGs serve as units for presenting demographic data and controlling block numbering. The dataset encompasses information on mobility patterns and visits made by users residing in CBGs inside NYC boundaries, spanning from January 2018 to December 2021.

It is important to note that mobility datasets derived from smartphone applications may contain sampling biases, since the observed population is limited to users who own smartphones, use applications included in the data panel, and have enabled location tracking. Therefore, the tracked devices should not be interpreted as a perfect random sample of the entire population. Such biases may vary across geographic, socioeconomic, and demographic groups. In this study, we address this issue in two ways. First, we evaluate the representativeness of the mobility panel by comparing tracked device counts with census population counts at different levels of aggregation. Second, we conduct a sampling-bias sensitivity analysis to examine whether the main temporal shifts in revealed shopping-location patterns are robust after excluding CBGs with the highest absolute sampling bias values.

The second dataset provides information about the places or points of interest (POIs) that the users visit. A POI commonly denotes a distinct geographic location or area that serves a specific purpose or provides particular amenities and attractions. These may include restaurants, gas stations, tourist attractions, transportation hubs, or notable landmarks. The dataset provided encompasses diverse information regarding the places, including their names, business categories, area measured in square footage, and geolocation coordinates. These details offer comprehensive insights into the characteristics and attributes of the included places.

It is important to note that both the mobility patterns and place datasets are geographically confined within the administrative boundaries of NYC. This encompasses 6,493 CBGs and 42,130 POIs. These datasets have been sourced from the SafeGraph COVID-19 Data Consortium, which provides curated POI and mobility data that has been used in previous studies of mobility and consumer behavior.

In addition, we integrate census data obtained from the United States Census Bureau (www.census.gov) into our analysis. This dataset provides 5-year estimates of crucial demographic information at the CBG level. It includes various demographic attributes such as median household income, education level, median age, and the racial composition of the residents. This census data helps us gain insights into the socioeconomic and demographic characteristics of the CBGs that are under examination. This information enhances our understanding of the contextual factors that may influence consumer behavior and revealed shopping-location patterns within these areas.

It is notable that previous research studies have highlighted the efficacy of these datasets in capturing consumer behavior from various viewpoints (Zhou et al., 2022; Sevtsuk et al., 2021; Fe and Sanfelice, 2022; Dolega et al., 2021; Bahrami, 2023). The richness of the data, which encompasses details on mobility patterns, visits, places, and demographic factors, offers a comprehensive perspective on customer shopping mobility behaviors. At the same time, considering the potential sampling limitations of mobile-location data, the results should be interpreted as patterns inferred from the observed mobility panel rather than as complete population-level measurements without uncertainty.

### 4.3 Analytical setting

In this section, we examine the specific context of retail stores in NYC, an urban center characterized by a high population (exceeding 8 million residents), advanced development, and modern infrastructure. NYC is renowned for its diverse demographic composition, encompassing residents from various backgrounds, and is home to numerous commercial brands striving to expand their market presence and gain a larger market share.

#### 4.3.1 Data preprocessing

Several previous studies have extensively examined and discussed the representativeness of SafeGraph mobility patterns at various levels of census statistical geographic areas, including counties, census tracts, and CBGs (Athey et al., 2020; Jay et al., 2020; Liang et al., 2020; Chang et al., 2021; Klise et al., 2021; Fe and Sanfelice, 2022; Li et al., 2023).

To improve the reliability of our model's input data and reduce the effect of extreme sampling observations, we implemented the following data preprocessing steps. These steps are intended to retain CBGs with consistent observations across the study period and to remove outlying CBGs in terms of sampling ratio and sampling bias. However, we do not interpret these steps as fully eliminating all possible sources of mobile-device sampling bias.

At the first step, to retain the robustness of the analyses, we filter and keep CBGs that: (1) have visit records during all 4 years (2018 to 2021); and (2) have demographic information available in the census dataset. From all 6,493 CBGs in the dataset, 6,078 of them have visit records over the course of the 4 years, but only 5,781 of them have complete demographic information available in the census dataset.

Subsequently, for each CBG, we calculate its sampling ratio and sampling bias using Equations 4, 5, respectively. The sampling ratio of *CBG*_*i*_, denoted as *SR*_*CB*_*G*__*i*__ in Equation 4, is determined by the count of devices tracked in *CBG*_*i*_ represented by *TD*_*CB*_*G*__*i*__ and the population size of *CBG*_*i*_ denoted by *N*_*CB*_*G*__*i*__. Additionally, Equation 5 defines the sampling bias of *CBG*_*i*_ as *SB*_*CB*_*G*__*i*__.


$$\begin{array}{l} SR_{CBG_{i}}=\frac{TD_{CBG_{i}}}{N_{CBG_{i}}} \end{array}$$ (4)

$$\begin{array}{l} SB_{CBG_{i}}=\left(\frac{TD_{CBG_{i}}}{\overset{5781}{\underset{j=1}{\sum }}TD_{CBG_{j}}}-\frac{N_{CBG_{i}}}{\overset{5781}{\underset{j=1}{\sum }}N_{CBG_{j}}}\right)\times 100 \end{array}$$ (5)

In the final step, we employ the Interquartile Range (IQR) method to identify and eliminate outlier CBGs based on their sampling ratio and sampling bias values. This method establishes the normal value range as *Q*1 − 1.5 × *IQR* to *Q*3+1.5 × *IQR*, where *Q*1 and *Q*3 denote the first and third quartile values, respectively. Any CBGs falling outside this normal range are considered outliers and subsequently removed from the dataset.

Following the removal of outliers using the IQR method, the resulting set of CBGs comprises more than 95% of the CBGs in NYC that had both visit records and demographic information. Specifically, out of the initial 5,781 CBGs, a total of 5,502 CBGs remain in the dataset. This high coverage indicates that the majority of CBGs, representing a significant proportion of the dataset, are retained for further analysis.

Furthermore, there is a correlation of 0.756 between the number of tracked devices in the mobility data panel and the corresponding populations of the remaining 5,502 CBGs. This correlation suggests that the preprocessed data retains a meaningful relationship between tracked device counts and population size. Nevertheless, representativeness is not uniform across all spatial scales and socioeconomic groups. Therefore, in addition to the descriptive representativeness assessment, we conduct a sampling-bias sensitivity analysis in which the main parameter-change results are recomputed after excluding the CBGs with the largest absolute sampling bias values. The results of this sensitivity analysis, reported in the Supplementary material, show that the main 2019–2020 and 2019–2021 patterns remain stable after these exclusions.

Within the places dataset, POIs are categorized using the North American Industry Classification System (NAICS) 6-digit sector codes. For the purpose of this study, we focus on the General Merchandise Stores sector (NAICS 452), which comprises Department Stores (NAICS 452210), Warehouse Clubs and Supercenters (NAICS 452311), and All Other General Merchandise Stores (NAICS 452319) (https://www.census.gov/naics/). These three categories collectively consist of 282 stores located in the NYC area. Although this sector spans a range of retail formats, from conventional department stores to warehouse clubs and general-merchandise/dollar-type chains, we pool them because they share a common NAICS classification and a comparable multi-category product assortment. To streamline the discussion, we refer to the stores belonging to this pooled category as “general merchandise and department stores” for the remainder of the paper, and we assess whether the main results are sensitive to this pooling in a format-level robustness check reported in Section 5.3.5.

More details on data representativeness assessment and sampling-bias sensitivity analysis are provided in the Supplementary material.

#### 4.3.2 Model building

Leveraging a multiplicative model as the foundation of our proposed approach, we are able to incorporate multiple attractiveness factors into the model concurrently. Taking into consideration the attractiveness factors identified in the existing literature (refer to Section 3) and the possibility of their extraction from our datasets, we have included a set of six variables in our analysis model. These variables should be interpreted as observable proxies for store accessibility, store characteristics, surrounding area characteristics, socioeconomic similarity, and prior revealed chain visitation, rather than as complete measures of all factors involved in customer decision-making.

1. Customer-Store distance (*D*_*ij*_): the customer-store distance is a measure of the relative convenience of accessing a specific store from the customer's location (Huff, 1964). In the Huff model tradition, the attractiveness of a store to a customer decreases as the distance between the customer's location and the store increases. We calculate the Haversine distance between the store location and the centroid of *CBG*_*i*_. The Haversine distance, which is the angular distance between two points on the Earth's surface, is considered a more realistic measure of store-CBG distance compared to the Euclidean distance.
2. Store area (*S*_*j*_): the incorporation of store size or floor area in the model is grounded on the principle that larger stores may provide a wider and more comprehensive assortment of products. The increased variety of choices and enhanced availability of products tends to be more enticing to consumers (Huff, 1964). This factor may have gained additional significance during the COVID-19 pandemic, as larger stores could provide more physical space for distancing and accommodate customers under capacity and distancing constraints. At the same time, store area should be interpreted as a proxy for store scale and potential capacity, not as a direct measure of product availability or operating conditions. The floor area for each store is available in the places dataset, and is provided in units of square feet.
3. Diversity of POI categories in a store's vicinity (*E*_*j*_): as underscored by economic models, the abundance and variety of amenities play a crucial role as attractiveness factors in drawing people to a specific geographical region (Ogawa, 1998; Glaeser et al., 2001; Adamson et al., 2004). Previous studies have demonstrated the effectiveness of incorporating these factors in extensions of the Huff model. They can serve as proxies for area attractiveness and the potential for multipurpose trips, as discussed in Section 3. Following Shannon's entropy formula (Shannon, 1948), we calculate the diversity value based on the business categories of the POIs within the CBG where store *j* is located. In statistical physics, Shannon entropy quantifies the number of possible arrangements or states within a system. When applied to urban amenities, it quantifies the number of different ways these amenities can be combined. As a result, it serves as a metric of the variety of experiences that customers can obtain from this diverse mix of amenities. In this study, POI diversity is therefore used as a proxy for the functional diversity of the store's surrounding area.
4. Count of POIs in a store's vicinity (*C*_*j*_): this factor serves as a measure of the abundance and availability of amenities within a geographic area (i.e., CBG). As described in item 3, this measure, along with the diversity of amenities, can serve as a proxy for the attractiveness of the area to a variety of customers and for the possibility of multipurpose trips. For each store *j*, we utilize the count of POIs within the CBG where the store is situated.
5. Store-customer CBG demographic similarity (*M*_*ij*_): this factor quantifies the socioeconomic similarity between customers' home CBGs and the neighborhoods where stores are situated. The inclusion of this factor in the Huff model has been grounded on the assumption that, given equal attractiveness and accessibility factors, customers may be more likely to select a store located in a neighborhood with socioeconomic characteristics similar to their own, compared to other alternatives (Bahrami et al., 2022). To calculate this measure, we construct a seven-dimensional vector representing the demographic attributes of individuals residing in each CBG. These attributes include education level (percentage of residents with a college degree or higher), income level (median household income), age (median age), and the racial composition of residents (percentage of White, African–American, Asian, and Hispanic/Latino individuals). Next, we calculate the cosine similarity between the demographic vectors of each customer CBG (*V*_*i*_) and store CBG (*V*_*j*_) pair.
6. Customer chain loyalty (*L*_*ij*_): the incorporation of this factor into the Huff model is founded on the notion that prior revealed visits to a particular chain or store can significantly influence the likelihood of selecting it for future shopping visits (Seenivasan et al., 2016; Bahrami et al., 2022). We define customer chain loyalty as the proportion of total visits made by customers from *CBG*_*i*_ to all stores within a specific chain or the brand that owns store *j* during a given time period. Therefore, this variable should be interpreted as a revealed chain-visitation measure at the CBG level, rather than as a direct psychological measure of loyalty. Because this measure is constructed from the same-period visits it is subsequently used to help explain, it is possible for a decline in the number of distinct chains or stores visited within a period to mechanically raise measured concentration on the remaining chains, independently of any genuine change in customer behavior. We assess this concern directly using a lagged specification of chain loyalty in Section 5.3.6.

Equation 3 serves as the foundation for presenting the closed-form expression of the utility function employed in our study case, which is represented by Equation 6.


$$U_{ij}=\frac{S_{j}^{\delta }\times E_{j}^{\eta }\times C_{j}^{\zeta }\times M_{ij}^{\theta }\times L_{ij}^{\xi }}{D_{ij}^{\beta }}$$ (6)

It's important to note that in our analysis of the two variables, namely: diversity of POI categories and the count of POIs near a store, we define the vicinity of a POI as the CBG where it is located. Although other measures like circular buffers or travel time and cost cutoffs are used in the literature to define a POI's vicinity, given the small size of CBGs in the United States, we find no significant differences between the results from these different methods. The results strongly correlate, with correlation coefficients around 0.8 depending on the buffer size. We chose the CBG as the geographic vicinity mainly because other key factors in our analysis, such as census attributes, are defined at the CBG level. This approach ensures consistency in geographic boundaries across our variables without significantly changing the results.

It should also be noted that these model variables capture observed store characteristics, surrounding area characteristics, accessibility, and prior mobility patterns, but they do not directly observe all operational conditions during the pandemic. For example, temporary changes in operating hours, capacity restrictions, or service availability could influence observed visits even among stores that remained active in the dataset. Therefore, the estimated model parameters are interpreted as revealed store-selection weights under the combined demand-side and supply-side conditions present during each year.

#### 4.3.3 Calibration of revealed store-selection weights

As introduced in Section 3.2, four different methods have been proposed and utilized for parameter calibration in the literature, namely: OLS, GLM, GWR, and PSO. Previous research using OLS, GLM, and GWR has generated mixed results in various settings and applications (Suárez-Vega et al., 2015; Beiró et al., 2018; Chong et al., 2020). However, recent studies have shown that the PSO technique outperforms the other three methods, with the calibrated parameters using PSO producing better fit to the input data in the Huff model (Suhara et al., 2021; Liang et al., 2020; Bahrami et al., 2022). Therefore, in this study, we employ the PSO technique for calibrating the revealed store-selection weights.

To determine the optimal parameter values, we choose Pearson's correlation coefficient (*r*) between the estimated visit distribution calculated by our proposed model (Equation 6) and the actual visit distribution of a CBG as the objective to be maximized. The calibration process aims to minimize the cost, which is defined as one minus the correlation coefficient between the estimated and actual visits for each CBG in each considered year ($1-r_{year^{t}}$). Therefore, the fitted parameters are selected based on their ability to reproduce the observed distribution of visits from each CBG to the available stores in a given year. These calibrated parameters are then used to compare revealed shopping-location patterns across years and socioeconomic groups.

#### 4.3.4 Normalization interval and PSO search space boundaries

The final step of this approach involves defining the upper end point of the normalization interval (Φ) and the limits of the search space for the PSO algorithm (*L*_*PSO*_ and *U*_*PSO*_). The objective in this step is to maximize the model fit by adjusting these variables.

It is worth noting that changing the value of Φ can influence the set of optimal calibrated parameters and shift candidate solutions in a six-dimensional space, where six corresponds to the number of calibrated parameters in the proposed model. Additionally, due to the metaheuristic nature of PSO, there is no guarantee of converging to the global optima (Freitas et al., 2020). Hence, setting the boundaries of the search space [*L*_*PSO*_, *U*_*PSO*_] is crucial to increase the likelihood of finding improved candidate solutions within our approach.

To maintain the increasing effect of the calibrated parameters on the utility value, we set the lower bound of the search space to 1 (i.e., *L*_*PSO*_ = 1), ensuring that the parameters have a positive impact on the utility. The upper bound of the search space (*U*_*PSO*_) is then determined to find the optimal combination with Φ that maximizes the model fit. This approach aims to enhance the efficiency of the PSO algorithm by reducing the size of the search space while maximizing the model fit.

At this stage, we encounter two main challenges. Firstly, due to the multistep design of our approach, it is not possible to define a closed-form mathematical expression that can map Φ and *U*_*PSO*_ to the output cost of the PSO algorithm that should be minimized. Thus, gradient-based optimization methods are not applicable. Secondly, given the large-scale design of our study, a six-dimensional PSO must be run once for each CBG in each of the four study years. Consequently, exhaustive search and brute-force approaches are computationally inefficient.

To overcome these challenges, we employ a simulated annealing algorithm (SA) (Kirkpatrick et al., 1983). SA is a metaheuristic optimization algorithm for approximating global optima in a discrete search space. This algorithm does not make any assumptions about the objective function and is problem-independent. These properties make it suitable for our needs as: (1) the variables Φ and *U*_*PSO*_ are predefined to take integer values in a discrete search space, and (2) there is no functional form that can relate these two variables (i.e., Φ and *U*_*PSO*_) with the PSO output cost values.

We assign the median of the PSO cost values computed for the CBGs in our study as the state cost, denoted as *CS*_*n*_. The objective of the SA algorithm is to minimize this state cost, which we represent as *CS*_*min*_. We then proceed to follow the steps outlined in Algorithm 1.

The algorithm begins by initializing the temperature of the initial state as *T* and setting a decay rate 0 < *k* < 1. We then assign random integer values to Φ and *U*_*PSO*_, representing the initial state as *S*_*o*_ = (Φ_*o*_, *U*_*PS*_*O*__*o*__). Next, we compute the corresponding state cost as *CS*_*o*_. We initialize the minimum initial cost as *CS*_*min*_ = *CS*_*o*_ and proceed with the algorithm.

Each state *S*_*o*_ in the algorithm has four neighbors, denoted as *S*_*o*1_, *S*_*o*2_, *S*_*o*3_, *S*_*o*4_, which are obtained by incrementing or decrementing the corresponding state's Φ or *U*_*PSO*_ value by one unit. The algorithm continues iterating until two conditions are met: (1) *T* reaches a small enough value where the transition probability *P*_*T*_ approaches zero. This is determined by comparing the value of *T* with a predefined small positive number (ϵ>0) at each iteration, and (2) the cost of the current iteration state is lower than the costs of all its neighboring states.

Algorithm 1 Using SA to find the optimal combination of Φ and *U_PSO_*. 
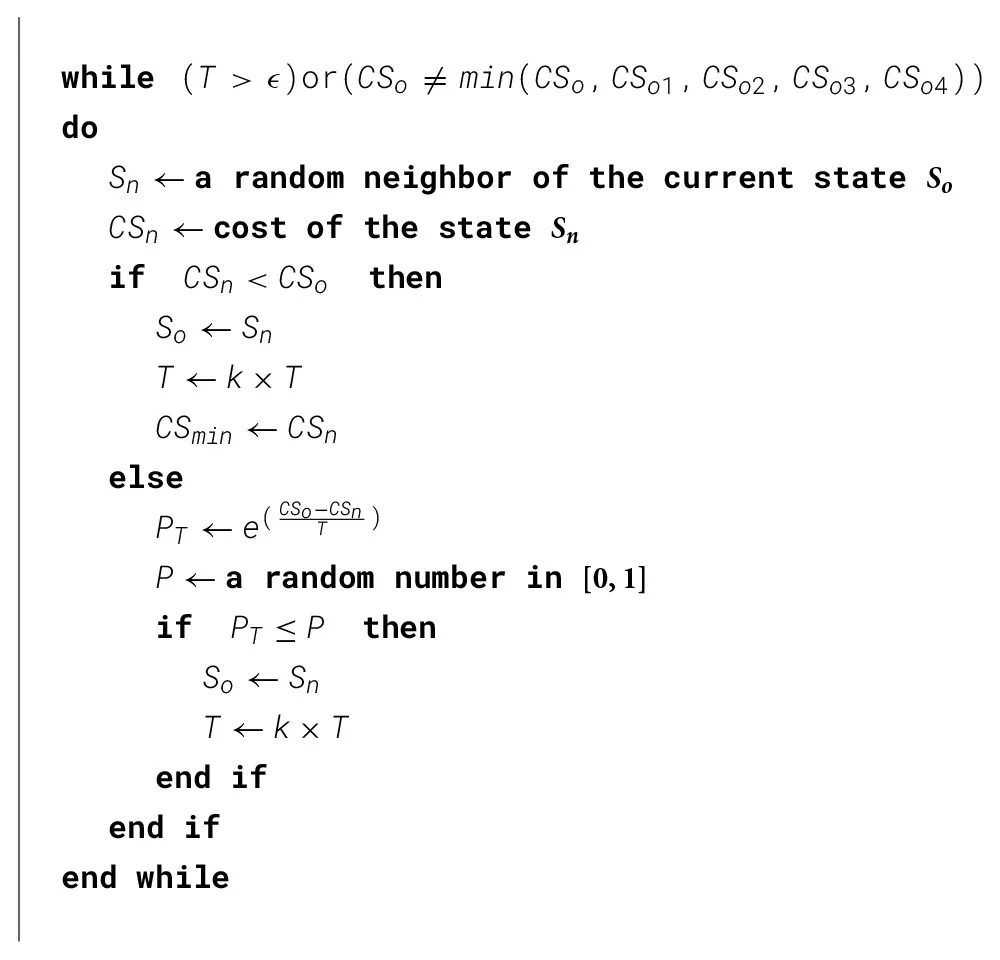

During each iteration of the algorithm, we maintain a record of the SA trajectory, which includes the states traversed and their respective costs. This allows us to compare the cost of the terminated state with the minimum cost observed throughout the iterations. Additionally, keeping a record of the states and their corresponding cost values reduces computational time in subsequent runs, as there is no need to recompute the cost for states that were previously traversed.

### 4.4 Overview of the proposed method

Figure 1 depicts a flowchart illustrating the schema of our approach, offering an overview of the inputs and analysis steps involved in calibrating the optimal set of store-selection weights.

**Figure 1:**
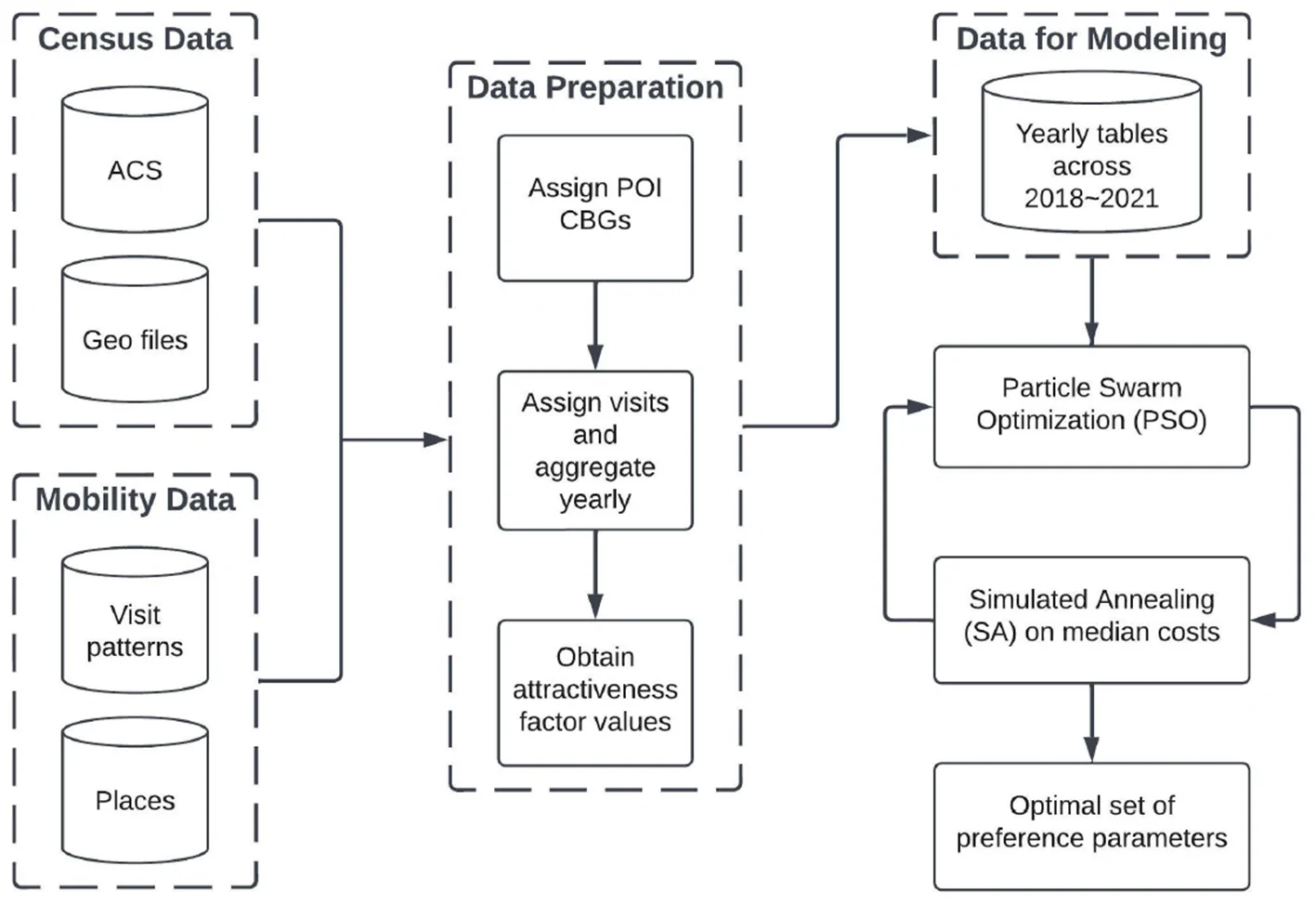
Parameter calibration process flowchart.

## 5 Results

To obtain the results, we fitted the model parameters at a CBG level on an annual basis from 2018 to 2021, resulting in six revealed store-selection weights for each CBG-year pair, corresponding to the six attractiveness factors incorporated into our model. The fitted model demonstrated significant correlations at the chain level for the years 2018, 2019, 2020, and 2021, with values of 0.964, 0.956, 0.742, and 0.544, respectively. These correlation values were computed by comparing the actual yearly visits to chains with the estimated visits obtained using the fitted parameters produced by PSO at its optimal point.

The lower model fit in 2020 and 2021 is expected, as mobility and shopping patterns during the pandemic and early recovery period were shaped by a more complex set of constraints than those observed during the pre-pandemic years. Although the stores included in this study were confirmed to be active in the dataset across the study period, temporary changes in operating hours, capacity restrictions, service availability, and other supply-side conditions could still affect observed visit patterns. The impact of these changes on the model's performance is likely limited to some extent because the model considers visit proportions rather than actual visit numbers. However, it is possible that an extreme decline in visit counts by residents within a CBG could affect the model's performance for the corresponding CBGs. To mitigate this issue, as explained in Section 4.3.1, we addressed the problem by including only CBGs that exhibited visits to such stores throughout the entire study timeframe.

It is important to note that the chain-level correlations reported above are aggregate diagnostics computed after summing estimated and actual visits to the chain level, and therefore average out much of the CBG-level noise in the underlying calibration. At the individual CBG level, where the PSO procedure is actually calibrated, model fit is both lower and more variable, especially in 2020 and 2021. Table 1 reports the distribution of CBG-level fit (defined as one minus the calibration cost, i.e., the Pearson correlation between the model-estimated and actual visit distribution for each CBG) across the 5,502 analytical CBGs for each year. Because our central non-reversion finding rests most heavily on the 2020 and 2021 parameter estimates, precisely the years in which CBG-level fit is weakest, we conduct an additional fit-stratified sensitivity analysis in Section 5.3.4 to assess whether the reported shifts in revealed store-selection weights are driven disproportionately by the subset of CBGs for which the model performs poorly.

**Table 1:** Distribution of CBG-level model fit by year.

Year	Mean	Median	P10	P25	P75	P90
2018	0.686	0.744	0.351	0.553	0.855	0.926
2019	0.694	0.748	0.370	0.570	0.859	0.927
2020	0.232	0.170	0.003	0.082	0.317	0.585
2021	0.165	0.142	-0.010	0.022	0.246	0.374

Fit is defined as one minus the PSO calibration cost (i.e., the Pearson correlation coefficient between the model-estimated and observed visit-share distribution for each CBG). Values are computed over the 5,502-CBG analytical sample described in Section 4.3.1.

The resulting calibrated parameters and their distributions were used to investigate the dynamics of revealed shopping-location patterns during the 4-year period. For each year, we used the parameter set that produced the highest correlation between the model estimated visits and actual visits from the data. The obtained results indicate that while there are no significant changes in the revealed store-selection weights from 2018 to 2019, with the outbreak of the COVID-19 pandemic, these parameters change significantly; therefore, in what follows, we focus primarily on understanding the effects of the pandemic on such mobility-based store-selection shifts.

### 5.1 The general effect of the COVID-19 pandemic on revealed shopping-location patterns

Figure 2 represents the yearly distributions of the calibrated store-selection weights from 2018 to 2021. Values do not change significantly from 2018 to 2019, while substantial changes are observed from 2019 to 2020 as well as smaller-scale shifts from 2020 to 2021. For instance, the calibrated weight associated with chain loyalty increased from 2019 to 2020, but its calibrated-value distribution in 2021 is similar to the one in 2020. On the contrary, while the importance of CBG-store distance decreased from 2019 to 2020, a similar trend was also observed between 2020 and 2021.

**Figure 2:**
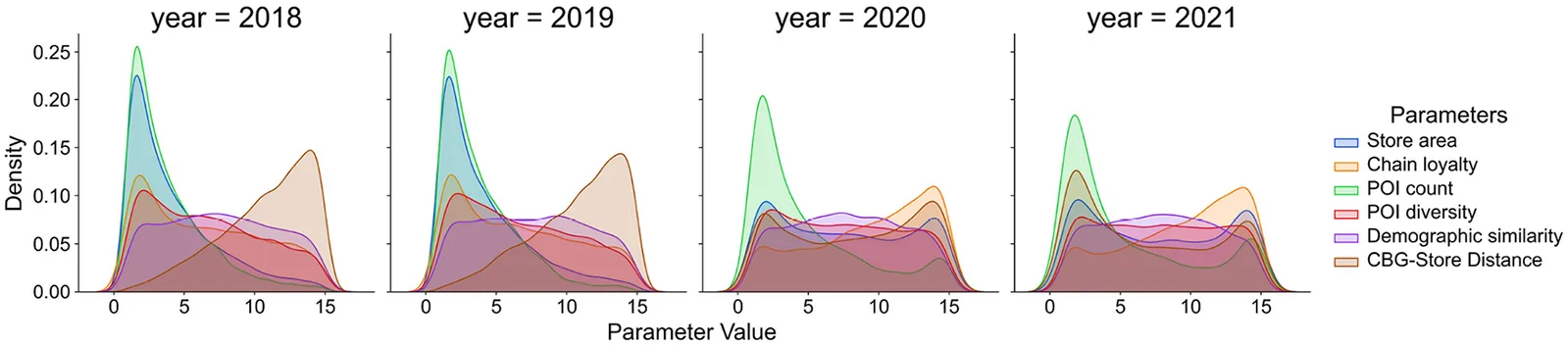
Yearly distribution of calibrated parameter values. Each subfigure depicts the distribution of store-selection weights for each of the six attractiveness factors in the corresponding year.

To validate the observations illustrated in Figure 2, we performed a Kolmogorov–Smirnov test (Massey, 1951) to compare the distributions of calibrated parameter values across consecutive years in the 5,502 CBGs where visits were captured consistently over the 4-year period. The resulting p-values can be found in Table 2. Under a two-sided 95% confidence level, there are no statistically significant changes in any of the calibrated parameter distributions between 2018 and 2019. However, with the onset of the COVID-19 pandemic in 2020, all six calibrated parameter distributions changed significantly at the 5% level. The change in demographic similarity was substantially weaker than the changes observed for the other parameters, with a p-value of 0.020 for the 2019–2020 comparison. Moreover, demographic similarity was not statistically significant in the 2019–2021 or 2020–2021 comparisons. In 2021, despite the commencement of the vaccination program, reopenings, and the gradual relaxation of various restrictions, most parameter-value distributions remained more similar to those observed in 2020 than to their pre-pandemic distributions. Therefore, 2021 is interpreted in this study as an early recovery and reopening period, rather than as a fully post-pandemic equilibrium.

**Table 2:** Kolmogorov–Smirnov test *p*-values in consecutive years.

Variable	2018~2019	2019~2020	2019~2021	2020~2021
Store area	0.91	9.407e-295^***^	7.222e-293^***^	9.835e-03^**^
Chain loyalty	0.851	5.294e-200^***^	2.990e-207^***^	0.312
POI count	0.439	5.150e-37^***^	5.652e-87^***^	3.965e-13^***^
POI diversity	0.574	4.311e-19^***^	1.240e-30^***^	2.140e-02^*^
Demographic similarity	0.734	2.020e-02^*^	0.063	0.946
CBG-store distance	0.622	5.138e-160^***^	0.000e+00^***^	4.195e-49^***^

This table provides the Kolmogorov–Smirnov test *p*-values for the distribution of each calibrated parameter in a pair of years. Statistical significance is denoted by: *p < 0.05, **p < 0.01, ***p < 0.001.

The comparison of the aforementioned findings with the temporal trends and global impact of the COVID-19 pandemic validates the ability of our model to capture shifts in revealed shopping-location patterns when residents select general merchandise and department stores. It also highlights the significant influence of the pandemic period on altering the store-selection patterns of New Yorkers when choosing a retailer. In essence, during crisis periods, observed mobility flows suggest that customers place different relative weights on store characteristics, chain visitation history, surrounding amenities, and customer-store distance.

Figure 3 illustrates the percentage change in mean (i.e., average) for each calibrated parameter from 2019 to 2020, 2019 to 2021, and 2020 to 2021. Notably, while the mean values of the demographic similarity parameter exhibit slight percentage changes in each year pair, the revealed store-selection weights indicate a greater emphasis on store area, chain loyalty, POI count, and POI diversity during the COVID-19 shock in 2020.

**Figure 3:**
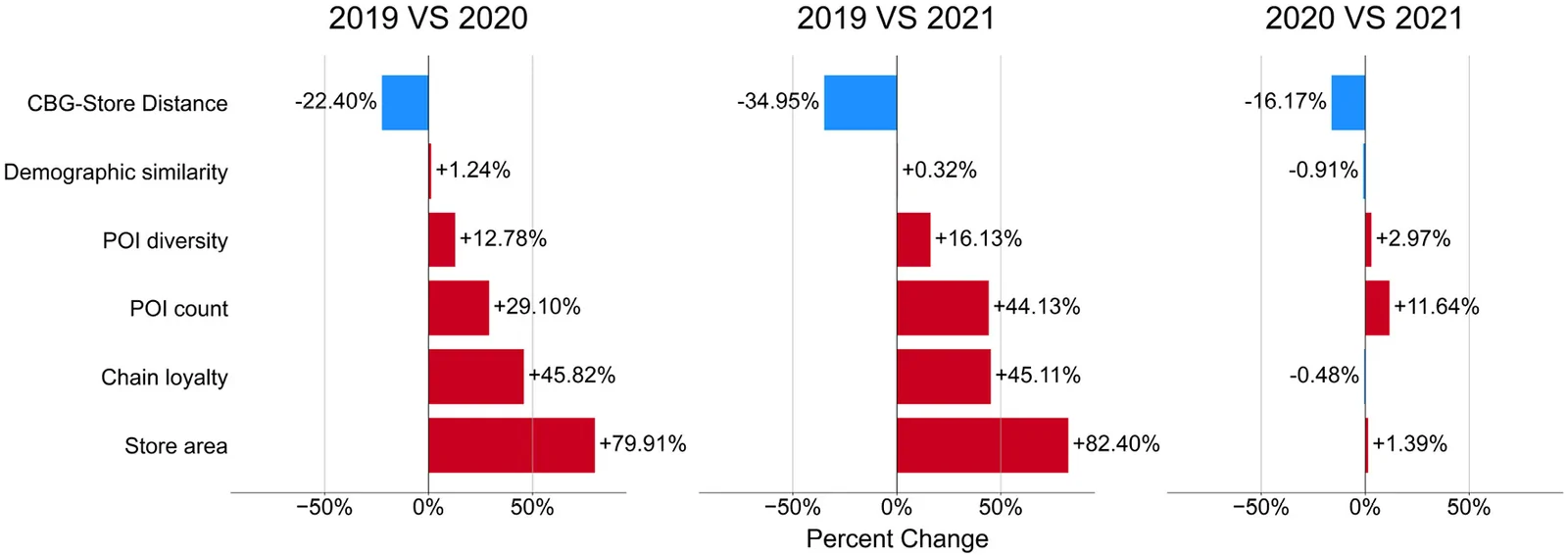
Percentage change in calibrated parameter values at the population level from 2019 to 2021. This figure shows the percentage change in the mean value of calibrated parameters between two different years. For example, in subfigure for 2019 VS 2020 the mean value of the CBG-store distance parameter in 2020 is 22.40% less than the same parameter value in 2019.

To elaborate, during the pandemic, the revealed weight associated with larger retail locations increased. This pattern may be related to the role of larger stores in providing more physical space, supporting distancing, and accommodating customers under capacity constraints. Additionally, the revealed weight associated with chain loyalty increased, suggesting that customers made fewer exploratory visits and concentrated more of their visits within previously selected chains. This behavior can be attributed to heightened search costs resulting from elevated health risks (Morozov et al., 2021).

Simultaneously, the revealed weights associated with stores located in areas with additional amenities and a more diverse range of businesses increased. This pattern was potentially related to the need to minimize the number of trips and satisfy multiple needs through multipurpose visits. Interestingly, the revealed weight associated with CBG-store distance decreased, suggesting that observed store-selection patterns became less distance-sensitive during the pandemic period.

However, the changes in the six calibrated parameters from 2020 to 2021 did not exhibit clear indications of reverting to their pre-pandemic distribution in 2019. This phenomenon suggests that the impact of the pandemic on revealed store-selection patterns toward general merchandise and department stores in NYC did not fully disappear during the early recovery period. In other words, within the available 2018-2021 observation window, New Yorkers' observed shopping mobility patterns remained different from their pre-pandemic baseline.

### 5.2 Heterogeneity among socioeconomic and demographic groups

The existing body of research has shed light on the unequal impact of the pandemic on various socioeconomic groups and the diversity of their response to COVID-19 containment measures (Almagro et al., 2023; Holtz et al., 2020; Chang et al., 2021; Liu et al., 2021; Heroy et al., 2021; Maleki et al., 2022; Gür, 2022). This emphasizes the need to explore the heterogeneity in how different communities have made shopping location choices during the pandemic and early recovery period.

In this study, we first focus on examining changes in calibrated parameter values from 2019 to 2020, as well as 2020 to 2021, specifically in communities where a single socioeconomic and demographic attribute ranks in the top 5% among all CBGs with records in NYC. This top 5% threshold is used to identify high-concentration communities with distinctive socioeconomic or demographic characteristics, making the comparison across groups more interpretable. However, because any fixed threshold may be considered somewhat arbitrary, we further examine the robustness of the main patterns using top 10% groups and additional multivariate socioeconomic structure checks based on PCA and K-means clustering, as reported in the Supplementary material and in Section 5.3. The socioeconomic attributes of interest include: education level (percentage of residents holding a college degree or higher), income level (median household income), age (median age), and racial composition of residents (percentage of White, African–American, Asian, and Hispanic/Latino residents). To facilitate the subsequent analysis, Table 3 presents the calibrated parameter values for the overall NYC population and the CBG groups ranking in the top 5% for each socioeconomic attribute in 2019.

**Table 3:** Calibrated parameter values for the population and top 5% socioeconomic communities in 2019.

Parameter	Population	Age	Education	Income	White	Black	Asian	Hispanic/Latino
Store area	4.179	3.644	2.674	3.141	3.436	5.119	3.915	5.164
Chain loyalty	6.435	6.489	8.247	8.127	6.678	7.058	5.027	5.839
POI count	3.779	3.925	4.077	4.14	4.2	3.712	4.26	3.898
POI diversity	6.725	7.473	7.574	7.894	7.524	5.48	7.301	6.578
Demographic similarity	7.882	8.34	8.516	8.261	8.087	7.573	8.142	7.879
CBG-store distance	10.651	10.453	10.65	10.412	10.23	10.783	10.373	11.134

The values provided in this table show the average values of the calibrated parameters for the six attractiveness factors at the NYC population level, as well as the seven CBG groups that rank in the top 5% of each single socioeconomic attribute as of 2019.

Figures 4, 5 display the percentage change in parameter values among the CBG groups ranking in the top 5% for each individual socioeconomic attribute in consecutive years. According to Figure 4, a noticeable trend is observed across all seven CBG groups: the revealed weight associated with CBG-store distance decreases, while the revealed weight associated with store area increases during the pandemic. Among the seven communities, except for the CBGs with White residents' proportions ranking in the top 5%, the count of POIs becomes increasingly important in the calibrated store-selection weights. The parameter associated with demographic similarity remains relatively consistent across all groups, while higher chain loyalty stands out among Asian residents, followed by Hispanics and residents in older age groups. This diversity in the change of the chain loyalty parameter among different groups may be attributed to variations in their perceived search costs.

**Figure 4:**
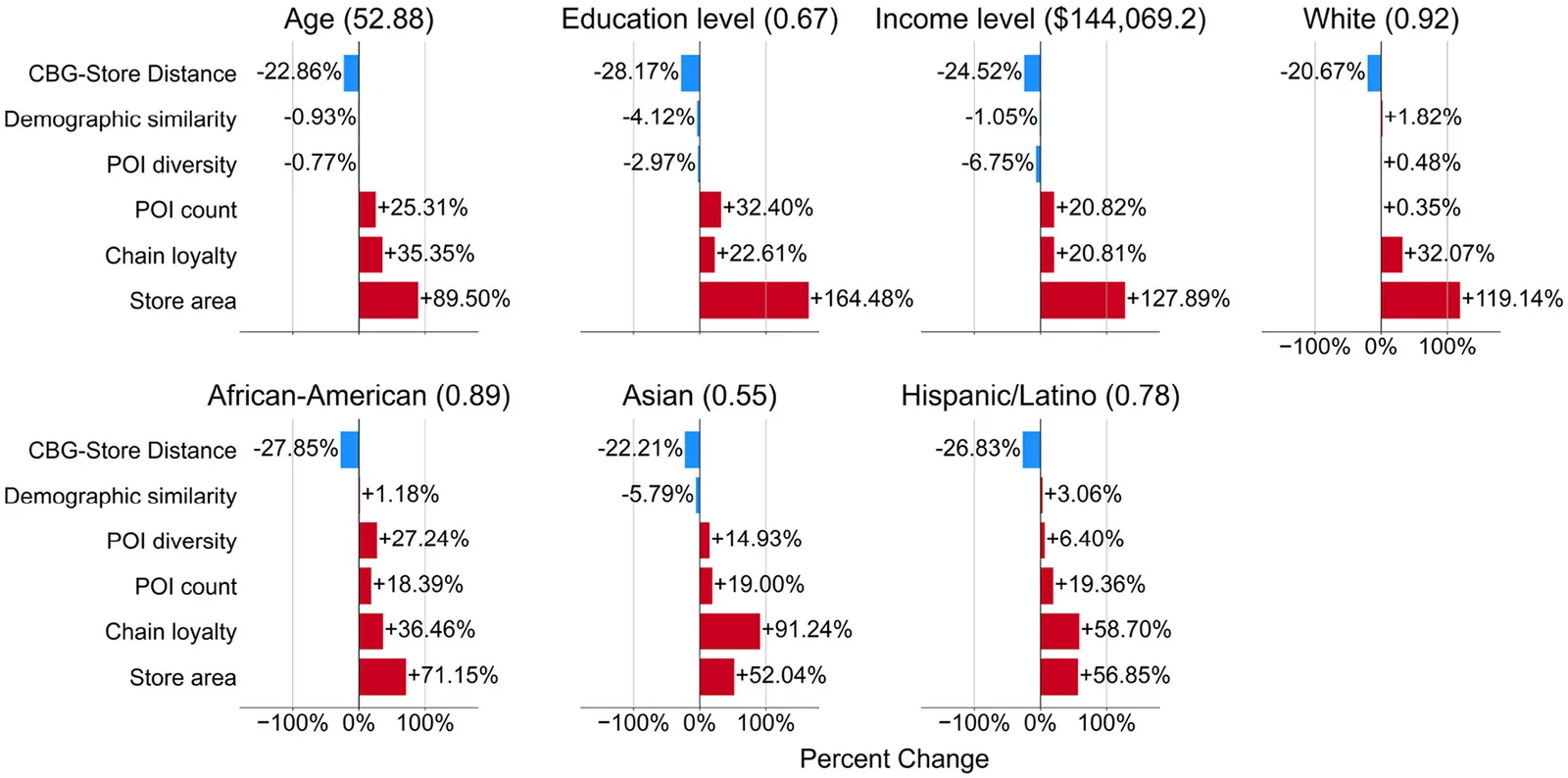
Percentage change in calibrated parameter values of top 5% socioeconomic communities from 2019 to 2020. In this figure, each subfigure presents how the value of each calibrated parameter changed from 2019 to 2020 among the top 5% communities based on different socioeconomic factors. The numbers in parentheses show the minimum value of each socioeconomic feature among the top 5% CBGs. For instance, the number in parentheses in subfigure for Education Level indicates that in the CBGs of the top 5% education level group, 67% of the residents hold a college degree or higher.

**Figure 5:**
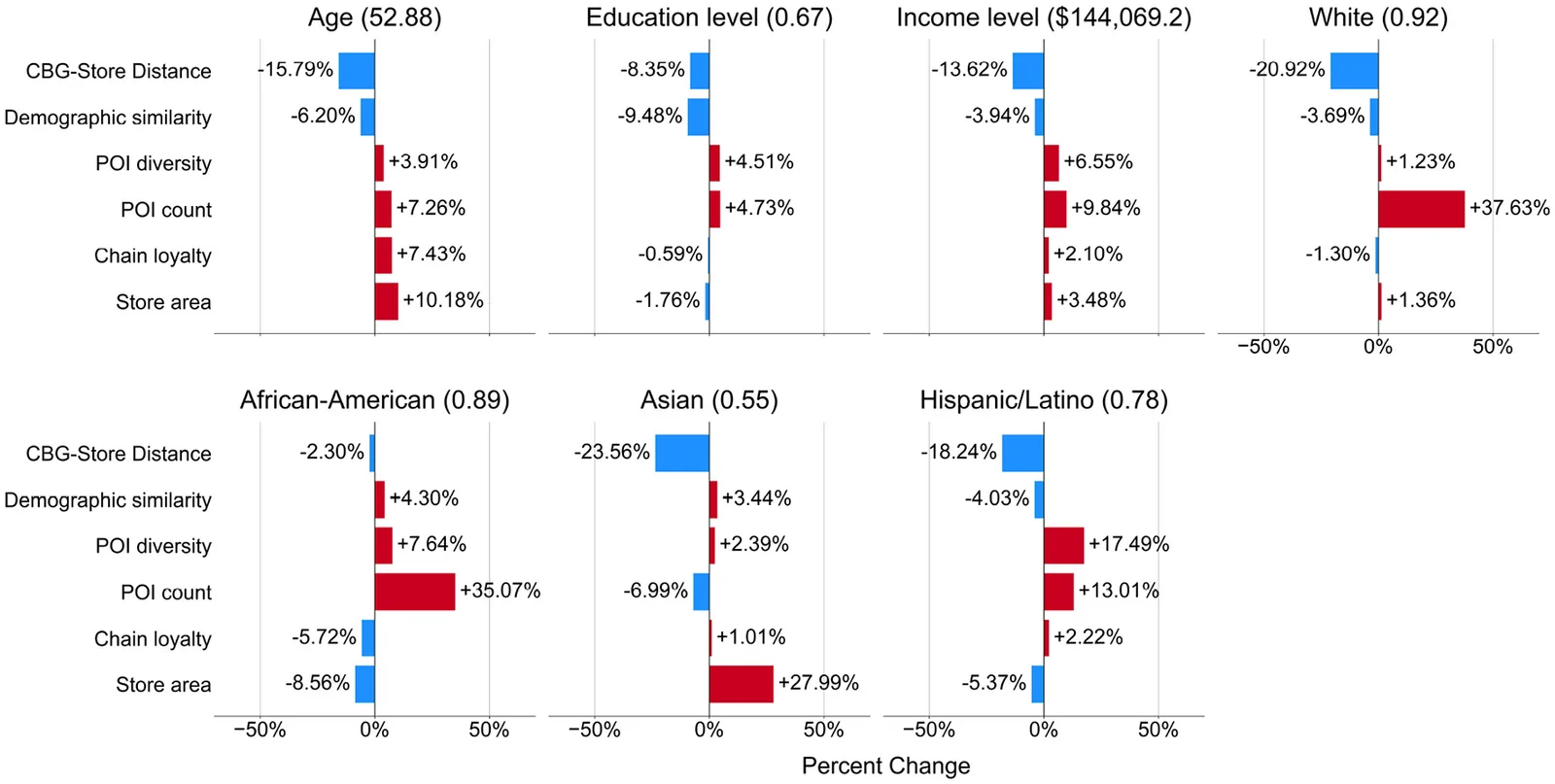
Percentage change in calibrated parameter values of top 5% socioeconomic communities from 2020 to 2021.

Although in 2021, as shown in Figure 5, the CBG groups ranking in the top 5% for each socioeconomic characteristic demonstrated a consistent trend of decreasing revealed weight associated with CBG-store distance, their calibrated weights for the other attractiveness factors showcased varied shifts. Consequently, it is necessary to analyze the shifts from the perspective of each individual community, and thus, in the following, we provide an explanation of the shifts observed in each community. Since there is a similarity in the trend of parameter changes among the top 5% communities in terms of education level, median age, and median income, we consolidate them and describe their revealed store-selection shifts under a unified perspective.

The top 5% CBGs in terms of age, income, and education level: the trends of parameter changes among these three communities exhibit a remarkable similarity, which can be attributed to notable pairwise correlations between age, income, and education level within communities. Specifically, their revealed store-selection patterns for general merchandise and department stores remained largely similar to those observed in 2020, with a slight increase in the calibrated weights associated with factors such as POI count, POI diversity, chain loyalty, and store area. It can be inferred that CBGs with higher age, income, and/or education levels, despite the gradual introduction of vaccination in 2021, did not fully return to their pre-pandemic store-selection patterns. Moreover, there was an even stronger emphasis on store area as the impact of the pandemic began to diminish within these communities.

The top 5% Asian resident proportion CBGs: within this group of CBGs, there is a continued emphasis on store area among residents, while the revealed weight associated with POI count decreased in 2021 after an initial increase from 2019 to 2020. Based on their consistent outlook toward the other five attractiveness factors, it can be inferred that residents in these CBGs showed revealed store-selection patterns more oriented toward larger general merchandise and department stores while placing less additional weight on surrounding amenities and commuting costs. This pattern may suggest stronger concentration of visits in chains or stores that satisfy their needs, regardless of the store's location.

The top 5% African–American resident proportion CBGs: the residents within this group of CBGs demonstrated a partial decrease in the revealed weight associated with store area, which had previously increased significantly during the pandemic. Instead, they showed a greater revealed weight associated with stores surrounded by a diverse range of facilities. Additionally, the distance from home factor became moderately less important for them.

The top 5% Hispanic/Latino resident proportion CBGs: the observed patterns of parameter change among the residents in this group are similar to those of CBGs within the top 5% proportion of African–American residents. It suggests that the environment of general merchandise and department store locations became more important in their revealed store-selection patterns, leading to similar shifts in the calibrated weights.

The top 5% White resident proportion CBGs: it is evident that the residents of CBGs in this group showed a higher revealed weight associated with the number of POIs in their selection of general merchandise and department stores. This shift in focus from distance suggests that as they gradually eased out of pandemic-related restrictions, the importance of proximity became less significant to them.

Along with the findings explained above, it is deduced that while some impacts brought by COVID-19 started diminishing during the early recovery period, some groups continued to show stronger revealed weights on a few parameters while placing lower weights on the rest of the aspects.

To get a better understanding of the direction in temporal shifts, we examine the change in changes of calibrated parameter values. For a calibrated parameter ω and a year *t* this value is denoted by Δω_*t*_ and computed as shown in Equation 7.


$$\begin{array}{c} \begin{array}{c} \Delta \omega_{t}=\left(\omega_{t}-\omega_{t-1}\right)-\left(\omega_{t-1}-\omega_{t-2}\right) \end{array} \end{array}$$ (7)

Figures 6, 7 show the average Δω_*t*_ values for the seven top 5% distinctive communities in each single socioeconomic feature, compared with the population average Δω_*t*_ values as of 2020 and 2021 separately. Since there are no significant shifts in calibrated parameters from 2018 to 2019, the Δω_*t*_ values for 2020 are very similar to change in the parameter values from 2019 to 2020, which makes the Figures 4, 6 convey a similar message. Therefore, in the following, we compare the differences in temporal store-selection patterns between communities and the general NYC population from the perspective of each attractiveness factor with a greater focus on values of Δω_*t*_ for 2021, as shown in Figure 7.

**Figure 6:**
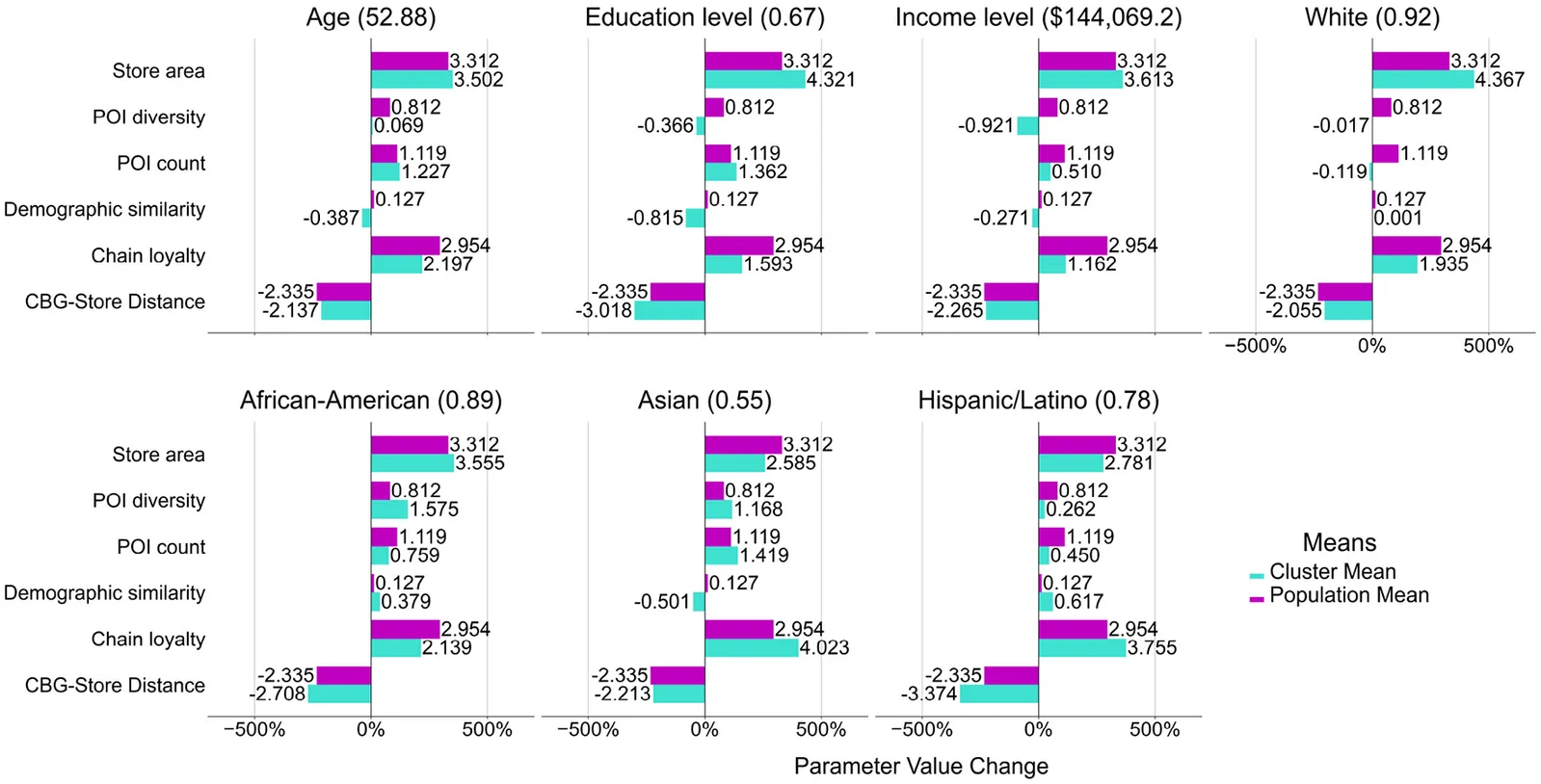
2020 Mean Δω_*t*_ Value Comparisons in top 5% socioeconomic communities and population. This figure shows the average change in changes of calibrated parameter values (Δω_*t*_) for the top 5% Socioeconomic Communities in each subfigure. The community Δω_*t*_ values are shown by blue bars and for comparison purposes the population level Δω_*t*_ values are shown by purple bars. Δω_*t*_ values are computed using Equation 7. The numbers in parentheses show the minimum value of each socioeconomic feature among the top 5% CBGs.

**Figure 7:**
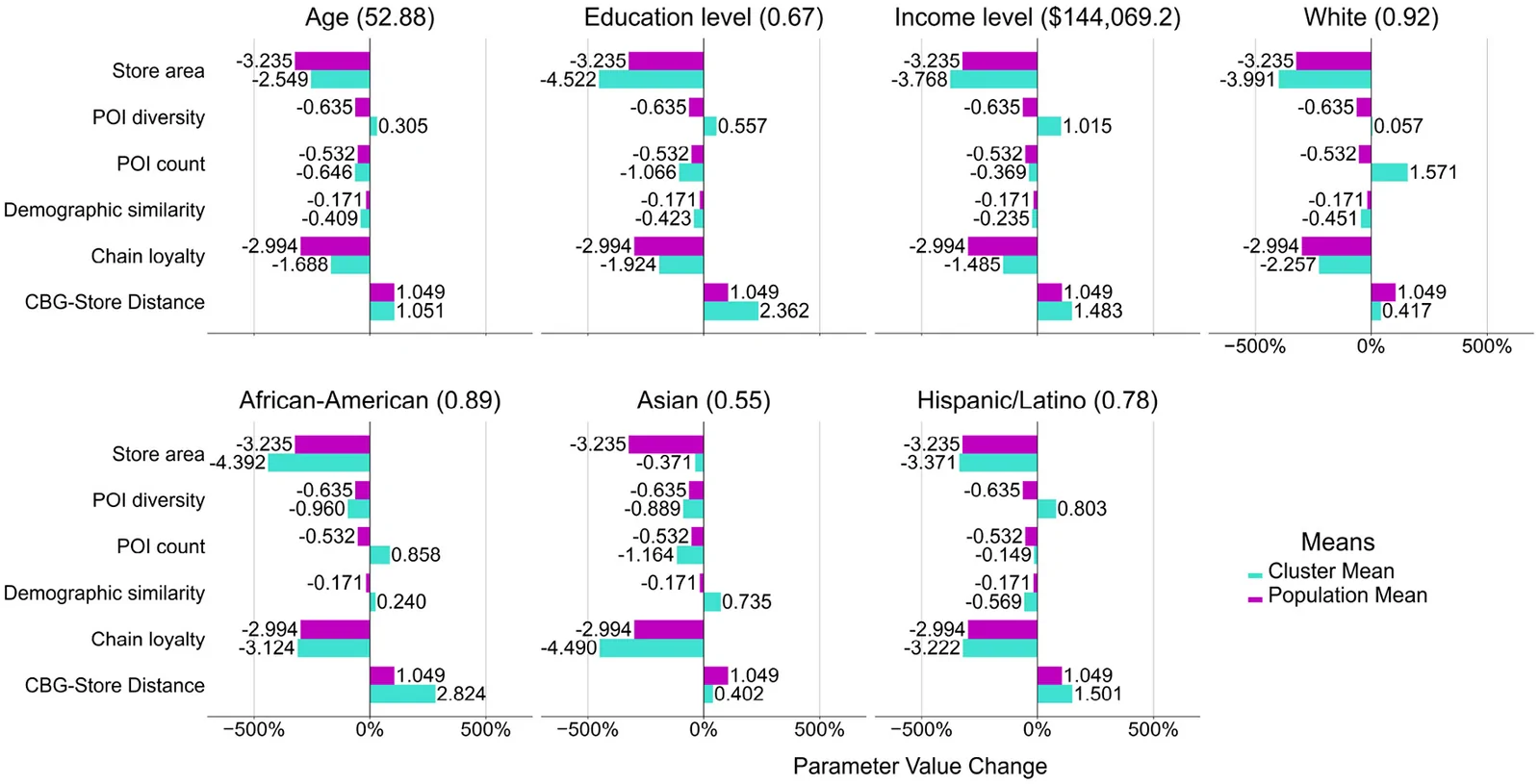
2021 Mean Δω_*t*_ value comparisons in top 5% socioeconomic communities and population.

Store area: The community with the top 5% Asian resident proportion showed limited change in the revealed weight associated with store area during both 2020 and 2021, indicating stronger concentration in their previously selected chains throughout the pandemic and early recovery period. In contrast, other communities, including those with higher education levels, median incomes, and older age groups, displayed an approximately 20% increased emphasis on store area during the pandemic. This suggests the presence of homogeneous subgroups with socioeconomic features where residents placed a higher revealed weight on store area amid the COVID-19 shock. Interestingly, in 2021, Asian resident CBGs showed relatively little additional change in the weight associated with store area compared to other communities, while communities with higher education levels and median incomes still showed a 20% increase in attention to store area. Additionally, CBGs with residents in the top 5% based on median age exhibited a significant 15% increase in attention to this feature, even greater than the initial increase when the COVID-19 pandemic started to spread. Conversely, African–American resident CBGs demonstrated a higher revealed weight for store area in their selection of general merchandise and department stores after the introduction of vaccination and relaxation of social restrictions.

Chain loyalty: it is observed that the revealed weight associated with chain loyalty among Asian resident CBGs grew faster during and after the pandemic shock, while higher income CBGs were most indifferent to this factor.

POI count: more educated, and African-American resident CBGs showed an increased revealed weight associated with POI count in the selection of general merchandise and department stores during COVID-19. Whereas, in 2021, the CBGs with a higher proportion of Asian residents, higher educated people, and those ranked higher based on the median age of their residents, favored stores with more POIs in their proximity.

POI diversity: African–American resident CBGs showed the largest increase in the weight associated with POI diversity both during the pandemic and during the early recovery period in 2021.

Demographic similarity: residents from higher education, and higher income CBGs were more likely to select stores located in neighborhoods with greater demographic similarities to them. This phenomenon suggests that the store-selection process may be partly shaped by the socioeconomic characteristics of the communities to which residents belong.

CBG-Store distance: during the early recovery period, CBGs with higher education levels, a higher African–American population and a higher age population increased their revealed weight associated with this factor more than the general population.

In addition, we compared the differences in temporal store-selection patterns between communities and the general NYC population, focusing on the direction of change from each attractiveness factor's perspective. For details about the analysis and results, please refer to the Supplementary material.

### 5.3 Validation and robustness checks

In this section, our objective is to validate and assess the robustness of the findings presented in Sections 5.1, 5.2. Firstly, we conduct a comparison between the model results and observed store-pair visit patterns. Additionally, we employ an unsupervised learning approach, specifically the K-means clustering method, to investigate the presence of heterogeneity in the response among clusters defined by combined socioeconomic characteristics. This unsupervised technique allows us to explore and analyze potential variations in revealed store-selection patterns without relying only on predefined labels or single socioeconomic attributes. We then provide additional robustness checks related to socioeconomic group definition, model fit and calibration confidence, format-level (NAICS) heterogeneity, the construction of the chain-loyalty measure, PSO estimator stability, sampling bias, clustering stability, and spatial autocorrelation. Finally, we employ a difference-in-differences method to estimate the impact of the pandemic on various aspects of revealed store-selection shifts among customers.

#### 5.3.1 Validating model results with observed store-pair visit patterns

To evaluate and validate the information derived from our experimental results in Section 5.1, we conduct an analysis of store visits over different years. Utilizing the model framework that considers multiple attractiveness factors concurrently, we compare stores with similar characteristics across all six factors, except for a difference in one specific factor. This approach enables us to simplify the comparisons and facilitate easier interpretation of the results.

Considering the basic statistics obtained from the attractiveness factors, it is observed that the store area exhibits the largest variance among the stores in our dataset. Consequently, we aim to select a pair of stores from each chain that fulfill certain criteria: they should be situated in CBGs with similar POI count and diversity, be geographically close to each other, or possess a comparable average distance to the population. By selecting stores from the same chain, we ensure that they share the same chain-visitation measure for that chain, leaving store area as the main variable attractiveness factor in this comparison. This approach allows us to examine whether the observed visit-share changes are consistent with the estimated increase in the revealed weight associated with store area.

According to our results in Section 5.1, during years 2020 and 2021, the calibrated parameter associated with store area increased when compared to 2019, while controlling for the other factors in the model. Hence, if two stores belonging to the same chain are located in CBGs with similar POI counts and diversities, the store with a larger floor area is expected to draw a higher share of customers in 2020 and 2021 in comparison to 2019, all else being similar.

Table 4 displays the observed examples for store pairs from the same chains that support the model results. In our dataset, the chains including Dollar Tree, Family Dollar Stores, Dollar General, T.J. Maxx, and Target own the highest numbers of stores in descending order. For each chain, we select one pair of stores with similar POI count and diversity in their vicinity (CBG), but evidently different scales of store size (i.e., floor area). Stores with a larger floor area have been shown to attract a higher share of visitors from 2019 to 2020 with the outbreak of COVID-19. During the transition into the early recovery period from 2020 to 2021, the magnitude of this shift became smaller in some cases, but the larger stores continued to draw relatively higher visit shares in most of the selected examples. Table 4 shows detailed information on the factors taken into account for each store. The last three columns provide the values of yearly visit counts and their corresponding visit share percentages in parentheses for the selected stores.

**Table 4:** Validation of model results using observed examples from store pairs within the same chains.

Store brand	Store ID	POI count	POI diversity	Area	Weighted distance	2019 visits	2020 visits	2021 visits
Dollar tree	s6g-kmk	24	2.556827168	4,147	8.374391	5,126(53.5%)	5,865(44.7%)	1,117(21.6%)
Dollar tree	s6f-w49	24	2.556827168	9,199	8.248515	4,448(46.5%)	7,266(55.3%)	4,047(78.4%)
Family dollar	s94-mhq	9	2.043191871	2,257	6.870538	3,605(50.2%)	3,989(48.8%)	2,027(38.6%)
Family dollar	s8x-3bk	10	2.025326221	14,301	7.136968	3,582(49.8%)	4,177(51.2%)	3,228(61.4%)
Dollar general	s6b-8vz	14	2.397895273	6,516	9.421959	3,606(50.8%)	3,963(40.1%)	3,254(50.6%)
Dollar general	wg9-ty9	14	2.205598359	8,080	8.226718	3,491(49.2%)	5,920(59.9%)	3,175(49.4%)
T.J. Maxx	s7m-h5z	50	2.902884061	901	7.028166	2,942(42.7%)	5,048(35.2%)	67(30.5%)
T.J. Maxx	wdh-swk	45	2.92211547	6,020	9.798352	3,956(57.3%)	9,288(64.8%)	153(69.5%)
Target	s8k-sdv	39	2.724920504	30,554	7.3432	6,750(61.7%)	5,043(36.7%)	2,542(33.8%)
Target	517E^*^	35	2.924102826	169,356	7.742251	4,185(38.3%)	8,712(63.3%)	4,972(66.2%)

^*^We noticed that three POIs with different Store IDs refer to the same location. Therefore, the information of those three POIs was aggregated and assigned to a new combined Store ID of 517E. The values in area and weighted distance columns are provided in square feet and miles, respectively. POI Diversity is computed using the Shannon entropy formula.

Although the observed store-pair examples support the results achieved by our proposed model, such insights may not be attainable through analyzing unidimensional descriptive statistics. To illustrate this limitation, let us consider the example of the importance of store area in shopping location decisions. Figure 8 showcases the cumulative distribution functions (CDF) of visit share based on store area for the years 2018 to 2021. If we examine the CDFs in Figure 8 and solely focus on store area as an isolated and independent attractiveness factor, one could mistakenly conclude that customers preferred small and medium-sized stores during the pandemic. However, such conclusions drawn solely from unidimensional analytics may fail to explain the reality and can be misleading. This occurs due to oversimplification of the customer decision process to a single dimension, while real-life decisions are influenced by complex interactions among multiple factors within a high-dimensional space. The subset of observed examples provided in Table 4 demonstrates that the revealed weight associated with store area increased in conjunction with the other attractiveness factors, challenging the notion that customers simply preferred small and medium-sized stores during the pandemic.

**Figure 8:**
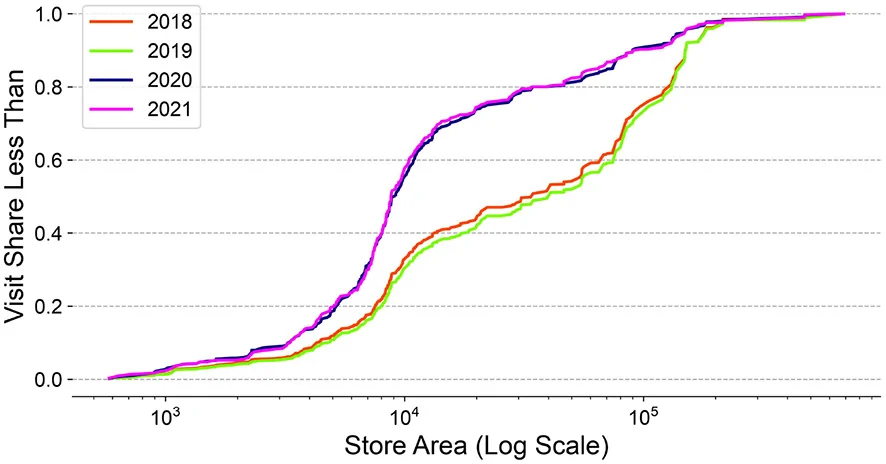
Cumulative distribution of visit frequency only by store area. Drawing conclusions based on these unidimensional illustrations may be misleading because such numerical results and figures ignore all other attractiveness factors and the interplay between those factors.

#### 5.3.2 Heterogeneity among clusters by combined socioeconomic features

In addition to the high-concentration socioeconomic group analysis in Section 5.2, where we explored communities that are more identifiable considering a single socioeconomic feature, we utilize an unsupervised clustering method in order to further investigate the spatial distribution of clusters based on combined socioeconomic features and validate our findings regarding the heterogeneity of revealed shopping-location patterns. This approach allows us to assess the robustness of our previous finding and gain insights into the variations in store-selection patterns among different clusters.

To achieve this, we employed the K-means clustering method to partition NYC's CBGs into five subregions, considering all seven socioeconomic attributes. The trained K-means model yielded a between-to-total sum of squares ratio (BSS/TSS) equal to 0.63, indicating a satisfactory separation of clusters. Table 5 presents the centroids of the clusters for each socioeconomic attribute, with the percentile indicated within parentheses alongside each centroid.

**Table 5:** *K*-means cluster centers by socioeconomic factors in NYC.

Variable	Cluster 1	Cluster 2	Cluster 3	Cluster 4	Cluster 5
Median age	39.889 (62.9%)	33.566 (29.8%)	37.662 (52.4%)	41.048 (68.5%)	41.356 (69.5%)
Education level	0.311 (69.6%)	0.117 (22.3%)	0.188 (43.4%)	0.226 (53.3%)	0.636 (93.4%)
Median income	$72969 (61.9%)	$41313 (22.4%)	$60493 (46.8%)	$63187 (50.3%)	$140215 (94.6%)
White	0.72 (77.1%)	0.26 (39.0%)	0.112 (20.4%)	0.294 (42.3%)	0.795 (83.7%)
African–American	0.056 (43.6%)	0.247 (64.6%)	0.749 (88.6%)	0.052 (42.5%)	0.039 (38.2%)
Asian	0.119 (63.2%)	0.058 (48.1%)	0.035 (37.8%)	0.509 (93.8%)	0.106 (60.7%)
Hispanic/Latino	0.194 (49.9%)	0.644 (87.7%)	0.144 (40.8%)	0.188 (48.9%)	0.095 (29.0%)

The values provided in parentheses show the percentile score of each attribute's centroid in the corresponding cluster.

Based on the centroids, the clusters can be interpreted as follows:

Cluster 1: predominantly White residents with average income levels.
Cluster 2: financially limited young residents, lowest income and education levels, with a majority being Hispanic/Latino.
Cluster 3: less educated, middle-aged residents with average income levels, and a majority being African–American.
Cluster 4: highest in terms of median age, mid-level education, and common-income residents with the most racial diversity, half are Asian.
Cluster 5: most educated and highest-income residents, with a majority being White.

The spatial distributions of the five clusters, as described earlier, are visualized in Figure 9.

**Figure 9:**
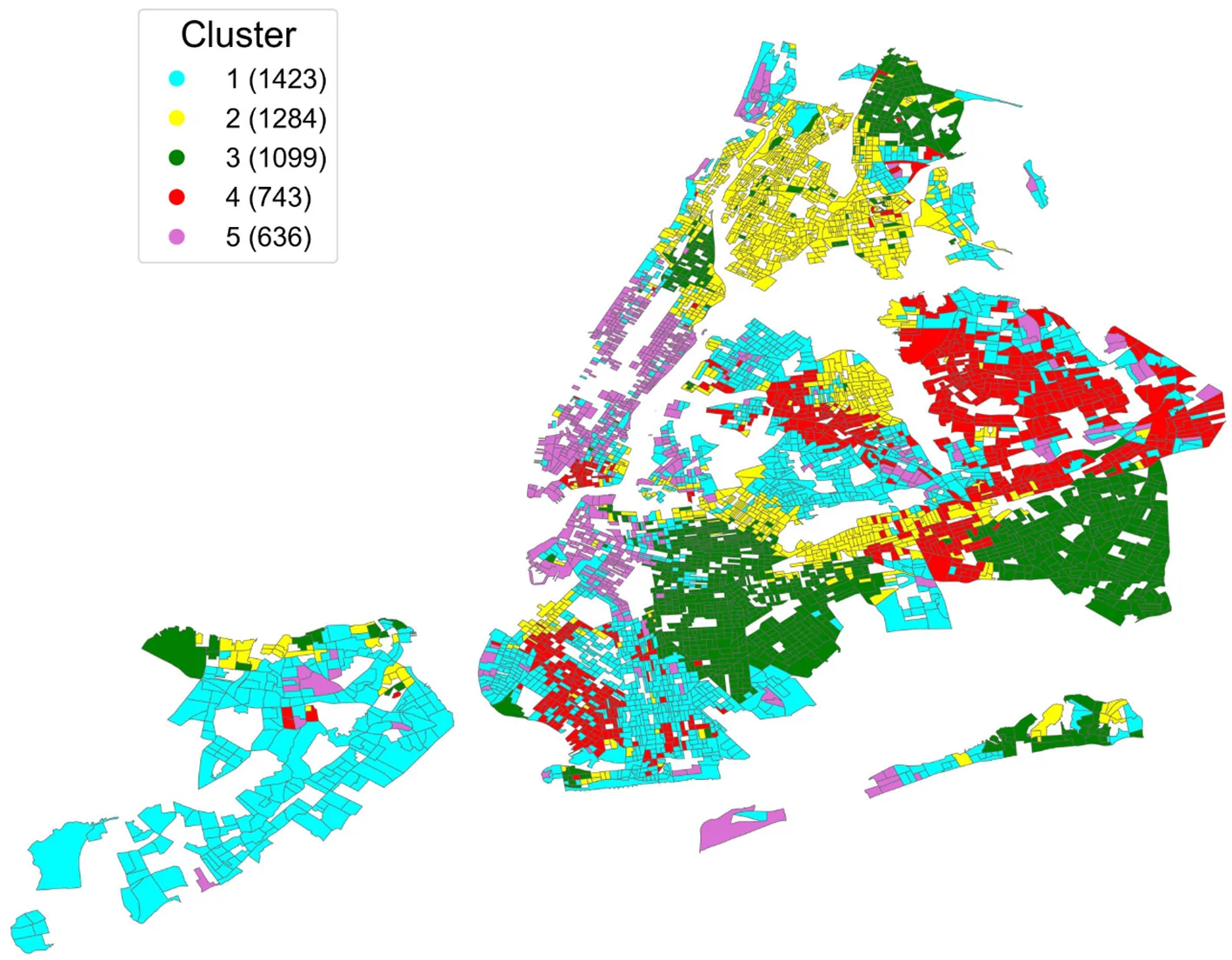
Spatial distribution of the five K-means clusters in NYC determined by combined socioeconomic attributes. In this figure, each polygon represents a CBG and the colors indicate the cluster a CBG belongs to. The numbers inside parentheses in the figure legend show the number of CBGs that belong to each corresponding cluster.

The collective trends in revealed store-selection weights across the five clusters from 2019 to 2020 and from 2020 to 2021 are illustrated in Figures 10, 11 respectively. During the transition from 2019 to 2020, as depicted in Figure 10, residents across all clusters exhibited a decreased revealed weight associated with distance and instead showed increased weights for stores with larger floor areas, and/or with a greater abundance and variety of nearby POIs. Furthermore, residents displayed an increased revealed weight associated with the chains they selected for in-person shopping.

**Figure 10:**
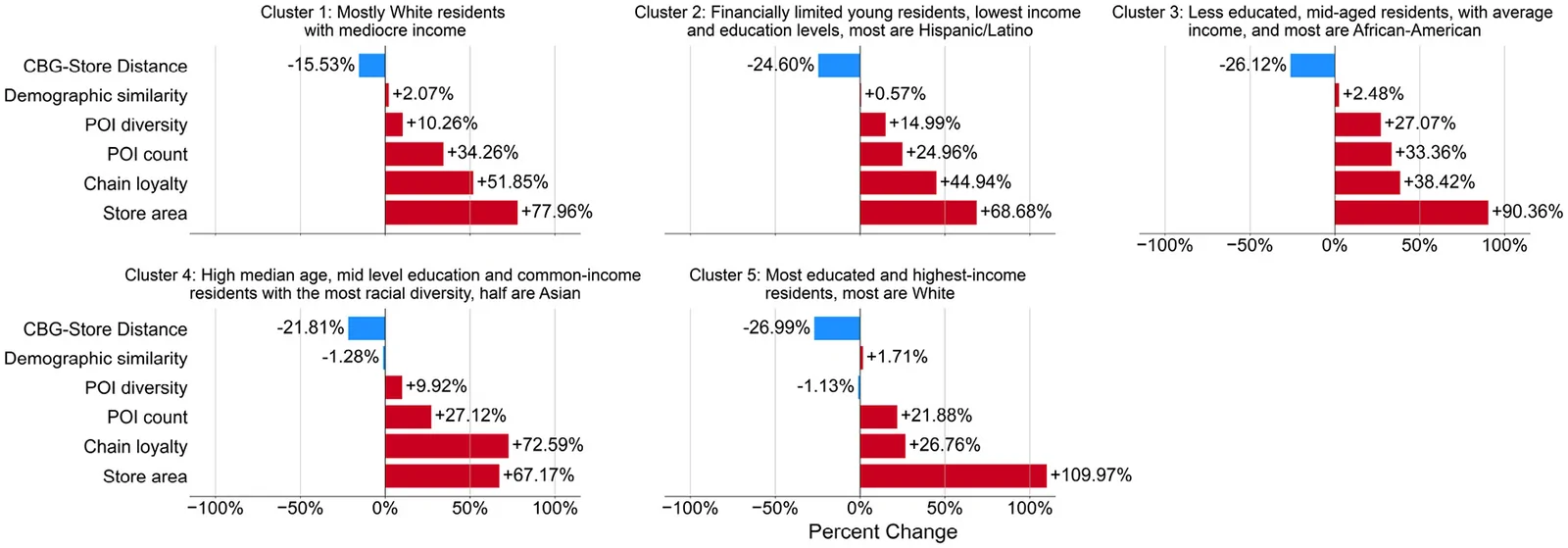
Percentage change in calibrated parameter values of K-means clusters from 2019 to 2020.

**Figure 11:**
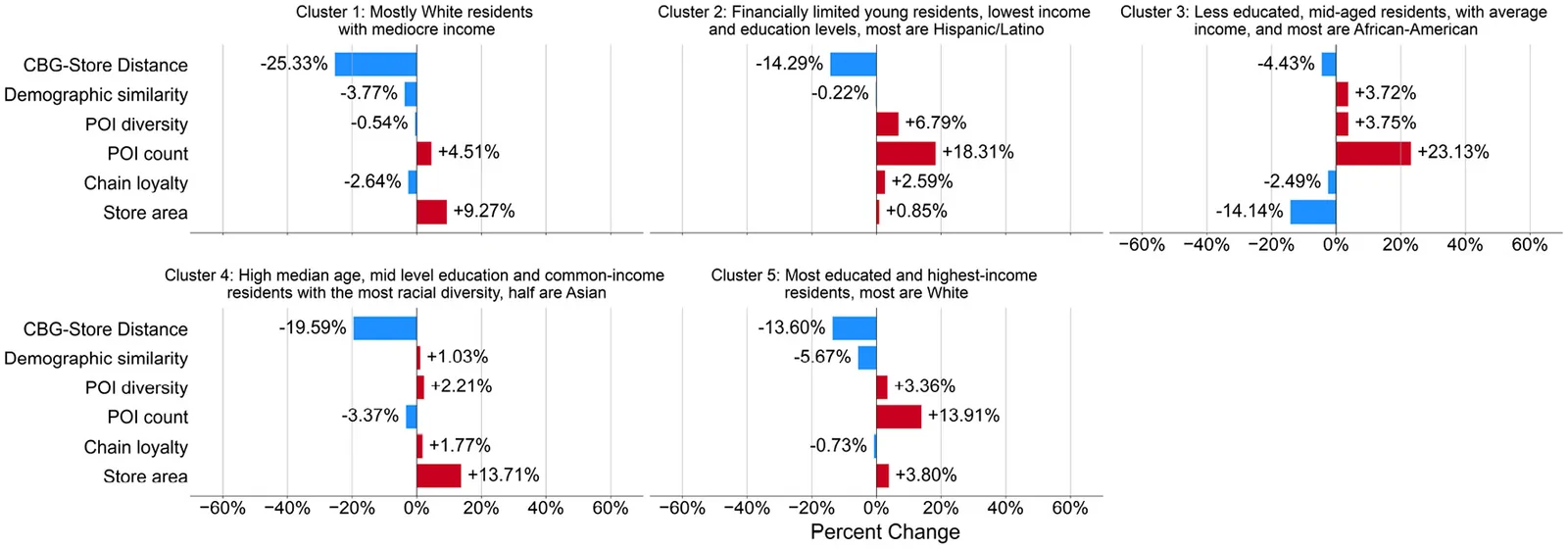
Percentage change in calibrated parameter values of K-means clusters from 2020 to 2021.

Figure 11 reveals that residents in Cluster 3 experienced a notable shift in their revealed store-selection weights between 2020 and 2021, as they significantly decreased the weight associated with store area. In contrast, the other communities demonstrated a reverse trend, placing increased weight on store area when visiting general merchandise and department stores in 2021. Additionally, all five communities showed a decreased weight associated with distance as a factor influencing their store-selection patterns. However, it is important to note that the shifts in the other attractiveness factors among the communities were relatively small and non-significant, despite the differences in directions.

For a more in-depth analysis and detailed results, please refer to the Supplementary material, where we compare the differences in temporal store-selection patterns between clusters and the general NYC population from the perspective of each attractiveness factor, with a particular emphasis on the direction of change.

Figures 12, 13 show changes in the value of Δω_*t*_ between 2020 and 2021. From 2020 to 2021, Δω_*t*_ changes reversed roughly among each community and the entire population. The earlier increase in the revealed weights associated with store size, chain loyalty, POI count, and POI diversity from 2019 to 2020 began to decline from 2020 to 2021, while the reduced role of CBG-store distance persisted in 2021. The magnitudes of the changes in 2021, nonetheless, are not as large as those in 2020. This occurrence confirms our earlier observation that following the outbreak, the revealed store-selection patterns of NYC residents changed from their pre-pandemic behavior and the impact of COVID-19 had not fully disappeared by the early recovery period in 2021.

**Figure 12:**
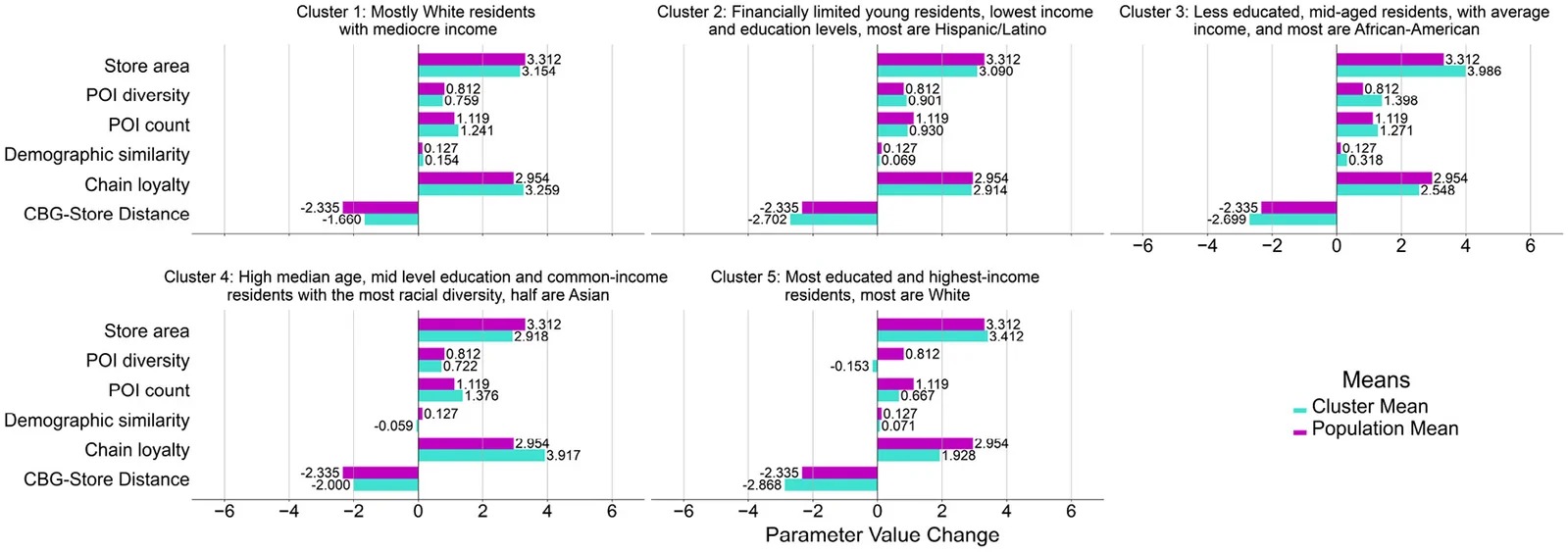
Mean Δω_*t*_ value comparisons in clusters by K-means as of 2020.

**Figure 13:**
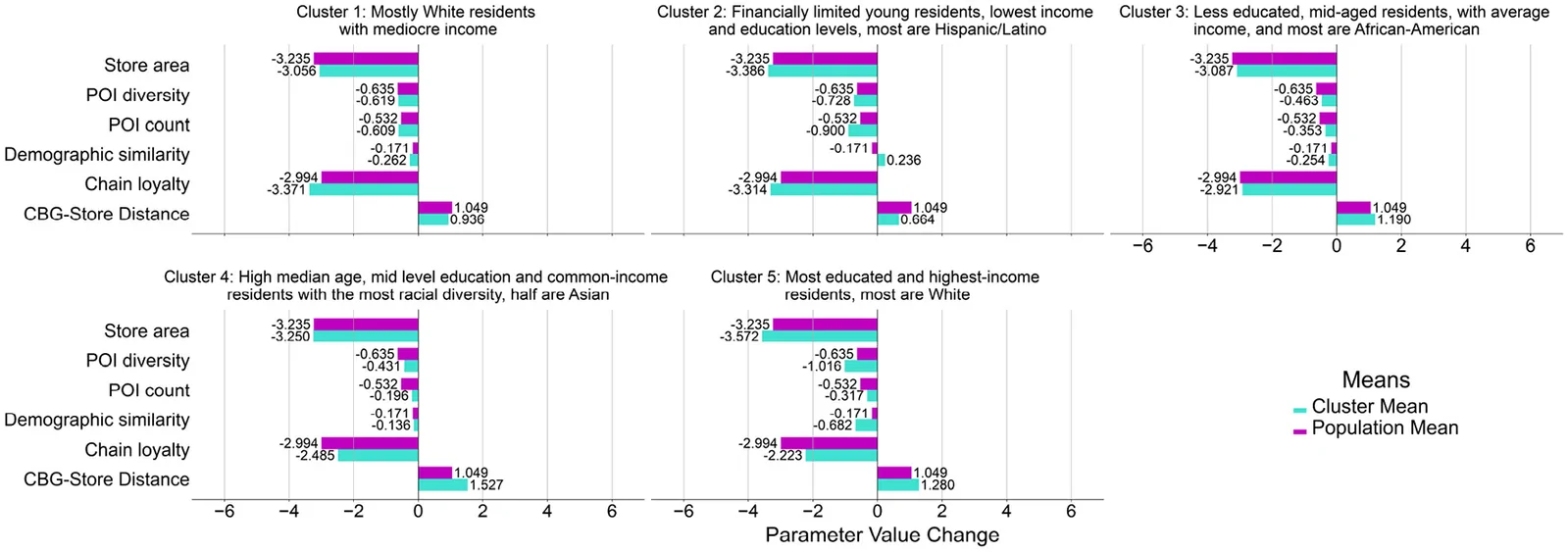
Mean Δω_*t*_ value comparisons in clusters by K-means as of 2021.

It has also been observed that changes in Δω_*t*_ due to store area, chain loyalty and CBG-store distance are apparently greater than those in POI count, POI diversity and demographic similarity in 2020, while in 2021 changes in Δω_*t*_ in CBG-store distance are similar to those of POI count and diversity. Consequently, it has been signaled that since the outbreak of COVID-19, the characteristics of stores and revealed prior chain visitation gained more importance relative to nearby commercial services and commuting cost. Overall, our findings using unsupervised clustering confirm that the pandemic response was heterogeneous among different communities.

To further examine the stability of the unsupervised clustering results, we reran the K-means procedure using standardized socioeconomic and demographic variables under 10 random seeds. The resulting partitions were highly stable, with pairwise Adjusted Rand Index values ranging from 0.995 to 1.000, with a mean value of 0.999 and a median value of 1.000. The inertia values were also highly stable across random seeds. These results indicate that the five-cluster solution is not sensitive to random initialization and that the cluster-level heterogeneity results are not driven by a particular K-means starting condition. Detailed results of this clustering stability analysis are reported in the Supplementary material.

#### 5.3.3 Additional socioeconomic robustness checks

As discussed in Section 5.2, the top 5% socioeconomic groups are used to identify high-concentration communities with distinctive socioeconomic and demographic characteristics. To assess whether the main heterogeneity results are sensitive to this threshold choice, we repeated the analysis using top 10% groups. The results remain consistent with the main findings. In particular, the 2019-2020 pandemic-shock pattern is robust: the revealed weight associated with CBG-store distance decreases across all top-decile groups, while the revealed weights associated with store area and chain loyalty increase across all groups. POI count also generally increases, while POI diversity and demographic similarity show weaker and more mixed patterns. The 2020–2021 comparison similarly suggests partial adjustment rather than full reversion to 2019. These findings indicate that the main results are not driven only by the specific top 5% threshold. The detailed top 10% results are reported in the Supplementary material.

We also conducted a PCA-based analysis of the socioeconomic structure of NYC CBGs to examine whether the observed heterogeneity is consistent with broader multivariate socioeconomic dimensions. The first three principal components explain 77.4% of the variation, while the first four explain 88.5%. The first component broadly captures an advantage or affluence dimension, with positive loadings on education, income, and White resident share, and negative loadings on Black and Hispanic/Latino resident shares. Additional components capture other demographic dimensions, including Black, Hispanic/Latino, and Asian population concentration. These results provide additional support that the heterogeneity observed in the main analysis is related to broader socioeconomic structure and is not only an artifact of a single threshold-based grouping rule.

#### 5.3.4 Fit-stratified sensitivity analysis

As shown in Table 1, model fit at the CBG level declines substantially in 2020 and 2021 relative to 2018-2019, confirming the decline already reported at the chain level in Section 5. Since our study's central non-reversion conclusion rests on the 2020 and 2021 parameter estimates, we test directly whether the reported shifts in revealed store-selection weights are an artifact of degraded calibration quality rather than a genuine change in observed shopping-location patterns.

We repeat the main percentage-change comparisons reported in Figure 3 after excluding the CBGs with the lowest calibration fit in the relevant post-shock year. For the 2019 to 2020 comparison we exclude the bottom 10%, 20%, and 25% of CBGs ranked by 2020 fit; for the 2019 to 2021 comparison we exclude CBGs ranked by 2021 fit. Table 6 reports the results. The direction of the main effects is unchanged in every specification, and in most cases the magnitude is similar to or larger than in the full sample. The revealed weight on store area increases by 78.6% in the full sample and by 77.4–82.6% after excluding the bottom 10–25% of CBGs by 2020 fit, chain loyalty increases by 43.5% in the full sample and by 44.0–44.4% in the fit-filtered samples, and CBG-store distance decreases by 22.5% in the full sample and by 17.9–21.3% in the fit-filtered samples. The 2019 to 2021 comparison shows the same pattern. POI count is the most sensitive to the fit filter where its estimated increase declines from 27.9% to 20.1–22.8% (2019 to 2020) and from 44.8% to 26.1–34.3% (2019 to 2021) after excluding low-fit CBGs, but remains positive throughout.

**Table 6:** Percentage change in calibrated parameter values, full sample vs. samples excluding the CBGs with the lowest model fit in the relevant post-shock year.

Parameter	Year-pair	Full sample	Excl. bottom 10% fit	Excl. bottom 20% fit	Excl. bottom 25% fit
Store area	2019–2020	+78.58%	+77.44%	+81.49%	+82.64%
Chain loyalty	2019–2020	+43.46%	+44.04%	+44.35%	+44.15%
POI count	2019–2020	+27.93%	+20.10%	+22.46%	+22.80%
POI diversity	2019–2020	+12.20%	+11.69%	+12.58%	+12.84%
Demographic similarity	2019–2020	+2.50%	+2.25%	+1.79%	+1.93%
CBG-store distance	2019–2020	–22.53%	–21.30%	–18.91%	–17.89%
Store area	2019–2021	+80.97%	+82.36%	+84.01%	+87.55%
Chain loyalty	2019–2021	+43.84%	+42.69%	+45.24%	+46.38%
POI count	2019–2021	+44.75%	+34.33%	+26.06%	+29.24%
POI diversity	2019–2021	+15.82%	+15.02%	+14.64%	+16.16%
Demographic similarity	2019–2021	+2.18%	+1.50%	+1.40%	+1.41%
CBG-store distance	2019–2021	–34.90%	–35.32%	–35.41%	–34.01%

CBGs are excluded based on their model fit (one minus PSO calibration cost) in the later year of each comparison. Quartile-based breakdowns and the 2020–2021 comparison are reported in the Supplementary material.

As a more stringent test, we split the CBGs into four quartiles of 2021 fit and repeat the 2019 to 2021 comparison within each quartile. The direction of change for store area, chain loyalty, POI diversity, and CBG-store distance is consistent across all four quartiles, including the lowest-fit quartile, indicating that the population-level shifts are not confined to, or driven by, the CBGs for which the model fits worst. The corresponding quartile-level results, together with the analogous 2019 to 2020 breakdown, are reported in the Supplementary material. The magnitude of the CBG-store distance decline is somewhat larger among lower-fit CBGs in the 2019 to 2020 comparison, which we interpret as a sign that lower calibration fit may inflate the magnitude, but not the direction, of the estimated distance effect.

Taken together, this analysis indicates that the main pandemic-shock and non-reversion findings are not an artifact of the CBGs with the weakest model fit, although the exact magnitude of the parameter shifts in 2020 and 2021 suggests somewhat more caution than the pre-pandemic estimates given the lower and more variable calibration fit in those years.

#### 5.3.5 Format-level (NAICS) sensitivity analysis

To assess whether the pooled results are driven by within-category heterogeneity between conventional department stores and other general-merchandise formats, we repeat the main percentage-change comparisons separately within each NAICS category defined in Section 4.3.1. We classify 242 of the 282 stores (86%) into Department Stores (NAICS 452210, *n* = 88) and General Merchandise Stores including Warehouse Clubs and Supercenters (NAICS 452319, *n* = 154) using SafeGraph's place and brand-level identifiers, which agreed on every directly comparable brand when cross-validated against one another. The remaining 40 stores (14%) could not be independently re-verified because their brands do not appear in either SafeGraph Places reference file available to us, while they are retained in the pooled analysis reported throughout this paper but excluded from this specific check. For each format, we identify CBGs with nonzero visits to that format's stores in all 4 years (*n* = 4,997 for Department Stores, *n* = 5,276 for General Merchandise) and draw a stratified random sample of approximately 450 CBGs per format, balanced by borough and household-income quintile, and re-calibrate the model independently for each format using the same PSO procedure and normalization settings as the main analysis.

Table 7 reports the results. The direction of change for store area, POI count, and CBG-store distance is consistent with the pooled analysis in both formats and both year-pairs. Chain loyalty, however, shows a format-dependent pattern: its pandemic-era increase is substantially larger among Department Stores (+33.9% in 2019 to 2020, +35.7% in 2019 to 2021) than among General Merchandise Stores (+3.4% and +4.9%, respectively), a pattern we examine directly in Section 5.3.6. Demographic similarity, already the weakest and least statistically robust parameter in the pooled analysis (Table 2), shows an inconsistent sign across formats and should continue to be interpreted with caution. We also find that the decline in model fit during 2020–2021 documented in Section 5.3.4 replicates independently within both format subsamples, indicating that this is a feature of the pandemic period itself rather than a result of pooling heterogeneous retail formats.

**Table 7:** Percentage change in calibrated parameter values: pooled sample vs. format-restricted subsamples.

Parameter	Year-pair	Pooled (*n* = 5,502)	Dept. stores 452210 (*n* = 448)	Gen. Merch. 452319 (*n* = 450)
Store area	2019–2020	+78.58%	+58.62%	+47.47%
Chain loyalty	2019–2020	+43.46%	+33.85%	+3.41%
POI count	2019–2020	+27.93%	+31.77%	+50.12%
POI diversity	2019–2020	+12.20%	+8.20%	+25.63%
Demographic similarity	2019–2020	+2.50%	–1.51%	+10.25%
CBG-store distance	2019–2020	–22.53%	–29.06%	–28.02%
Store area	2019–2021	+80.97%	+54.15%	+44.45%
Chain loyalty	2019–2021	+43.84%	+35.70%	+4.91%
POI count	2019–2021	+44.75%	+61.87%	+96.24%
POI diversity	2019–2021	+15.82%	+9.51%	+10.11%
Demographic similarity	2019–2021	+2.18%	–5.82%	+6.81%
CBG-Store Distance	2019–2021	–34.90%	–35.80%	–39.58%

Dept. Stores and Gen. Merch. columns are calibrated independently on stratified random subsamples of CBGs restricted to each format's store choice set. The 2020–2021 comparison is reported in the Supplementary material.

We conclude that the pooled results for store area, POI count, and distance are not an artifact of within-category heterogeneity between department-store and general-merchandise/warehouse-club formats, while the chain-loyalty finding is more format-specific than the pooled estimate alone conveys.

#### 5.3.6 Lagged chain-loyalty sensitivity analysis

We test the potential mechanical-concentration concern raised for the chain-loyalty measure (Section 4.3.2, item 6) directly, in two steps.

First, we examine the raw visit data underlying the loyalty measure. The correlation between a CBG's change in total visit volume and its change in brand-level concentration is weak (Pearson *r*≈0.03 to 0.20 across our specifications), while the correlation between the change in the number of distinct chains visited and the change in brand-level concentration is considerably stronger and consistently negative (*r*≈−0.29 to −0.61). This indicates that the mechanism of concern operates more precisely through a narrowing of exploratory breadth (visiting fewer distinct chains) than through the sheer decline in trip volume per se.

Second, and more directly, we re-calibrate the model using a lagged specification of chain loyalty: for each target year, the loyalty input is computed from the prior year's visits rather than the target year's own visits, so it cannot have been mechanically shaped by that year's own pandemic-era trip collapse, while the outcome being fit remains the true observed visits for the target year. We validate this procedure with a placebo check on the 2018 to 2019 transition, a period already shown to have no significant parameter shift (Table 2): substituting 2018 loyalty for 2019 loyalty when fitting the true 2019 visits changes every one of 18 parameter/sample combinations examined by less than 10% (maximum absolute difference 9.0%), confirming that the lagging procedure itself introduces no material distortion.

Table 8 reports the results. At the population level, the chain-loyalty increase substantially survives the lagged specification for the comparison underlying our central non-reversion conclusion: the 2019 to 2021 increase is 43.8% under the original (same-period) specification and 40.3% under the lagged specification, retaining approximately 92% of the original effect. The acute pandemic-shock comparison (2019 to 2020) retains a smaller but still substantial share (43.5% to 24.8%, approximately 57% retained).

**Table 8:** Chain-loyalty percentage change under the original (same-period) vs. lagged specification.

Sample	Year-pair	Same-period (unlagged)	Lagged	Share of effect retained
Pooled	2019–2020	+43.46%	+24.82%	~57%
Pooled	2019–2021	+43.84%	+40.30%	~92%
Dept. Stores 452210	2019–2020	+33.85%	+4.10%	~12%
Dept. Stores 452210	2019–2021	+35.70%	+19.93%	~56%
Gen. Merch. 452319	2019–2020	+3.41%	–1.68%	– (sign reversal)
Gen. Merch. 452319	2019–2021	+4.91%	–3.04%	– (sign reversal)

“Lagged” substitutes the prior year's chain-loyalty input while fitting the true target-year visits. Placebo check and full six-parameter lagged results are reported in the Supplementary material.

However, disaggregating by the format categories introduced in Section 5.3.5 reveals that this population-level pattern masks considerable heterogeneity. Within Department Stores, most of the 2019 to 2020 chain-loyalty increase does not survive the lagged specification (33.9% to 4.1%), indicating that for this format, in this specific acute-shock year, the mechanical-concentration mechanism largely accounts for the original estimate. Within General Merchandise stores, no positive chain-loyalty effect survives lagging in either year-pair; both comparisons turn slightly negative under the lagged design (2019 to 2020: 3.4% to –1.7%; 2019 to 2021: 4.9% to –3.0%), consistent with this format already exhibiting high pre-pandemic brand concentration and therefore comparatively little room for a genuine further increase.

We interpret this as a partial, format- and year-specific confirmation of the mechanical-concentration concern: it is well-founded for the acute 2020 shock within the department-store format specifically, while the population-level, multi-year non-reversion conclusion, which does not rest on that estimate alone, is comparatively robust to it.

#### 5.3.7 PSO estimator-stability analysis

To assess the stochastic stability of the PSO-calibrated parameters, we first confirm that our calibration procedure is reproducible under a fixed random seed. We then draw a representative stratified random sample of 300 CBGs, balanced by borough and household-income quintile, and re-calibrate the model for all 4 years under ten different random seeds (12,000 individual PSO runs in total), holding the sample fixed so that any variation reflects estimator stochasticity alone.

Table 9 summarizes the results. The direction of change for store area, chain loyalty, POI count, and CBG-store distance agrees across all ten seeds, for both the 2019 to 2020 and 2019 to 2021 comparisons, and none of their ranges cross zero. POI diversity agrees in direction for 8 of 10 seeds in both comparisons. Demographic similarity, already the weakest and least statistically robust of the six parameters (Table 2), shows the least stable pattern: only five of 10 seeds agree with the majority sign for 2019 to 2020, and six of 10 for 2019 to 2021.

**Table 9:** PSO estimator-stability summary: percentage change in mean parameter value across ten random seeds (300-CBG stratified subsample).

Parameter	Year-pair	Mean	Std	Min	Max	Directional agreement
Store area	2019–2020	+68.15%	14.04	+42.86%	+88.07%	10/10
Chain loyalty	2019–2020	+29.86%	11.82	+7.29%	+46.86%	10/10
POI count	2019–2020	+24.87%	10.20	+8.63%	+39.88%	10/10
POI diversity	2019–2020	+11.18%	6.82	–1.16%	+18.25%	8/10
Demographic similarity	2019–2020	+2.77%	6.28	-4.12%	+16.41%	5/10
CBG-store distance	2019–2020	–23.61%	3.49	–28.72%	–18.90%	10/10
Store area	2019–2021	+78.44%	17.98	+46.28%	+109.99%	10/10
Chain loyalty	2019–2021	+35.19%	12.43	+14.55%	+58.66%	10/10
POI count	2019–2021	+52.60%	12.67	+29.72%	+69.34%	10/10
POI diversity	2019–2021	+12.36%	11.71	–7.19%	+27.16%	8/10
Demographic similarity	2019–2021	–0.36%	9.53	–10.72%	+24.08%	6/10
CBG-store distance	2019–2021	–35.18%	3.98	-42.86%	–29.61%	10/10

Directional agreement reports the number of the ten seeds whose sign of change agrees with the majority sign, out of 10.

While the direction of the main effects is highly stable, point-estimate magnitudes carry meaningful seed-to-seed variability at this subsample size. For example, the 2019 to 2020 chain-loyalty percentage change ranges from approximately 7% to 47% across the ten seeds (mean 29.9%, standard deviation 11.8%). We therefore report the specific percentages elsewhere in this paper as representative point estimates from a stochastic calibration procedure rather than as fixed, deterministic quantities, while treating the direction and qualitative pattern of the findings, which this analysis shows to be highly robust, as the primary basis for our conclusions. Individual CBG-level parameter estimates are considerably noisier still (median coefficient of variation across CBGs of 30–55% depending on the parameter and year), which supports our approach of drawing conclusions from population- and group-level aggregates rather than from individual CBG point estimates. Full results, including cross-CBG rank-correlation diagnostics across seeds, are reported in the Supplementary material.

#### 5.3.8 Sampling-bias sensitivity analysis

Considering the potential sampling bias in mobile-location datasets, we further assessed whether the main parameter-change results were sensitive to CBG-level sampling bias in the SafeGraph panel. To do so, we repeated the parameter-change analysis after excluding the CBGs with the highest absolute sampling bias values. The main 2019–2020 shifts remained highly stable. Store area increased by 78.6% in the full sample and by 77.7% and 78.1% in the two bias-filtered samples. Chain loyalty increased by 43.5%, 44.2%, and 44.3%, respectively. POI count increased by 27.9%, 28.1%, and 28.3%, while CBG-store distance decreased by 22.5%, 22.3%, and 22.4%.

The 2019–2021 comparison also remained stable after excluding the most sampling-biased CBGs. Store area increased by 81.0% in the full sample and by 80.3% and 80.8% in the two bias-filtered samples. Chain loyalty increased by 43.8%, 44.1%, and 44.0%, while CBG-store distance decreased by 34.9%, 34.6%, and 34.1%. These results suggest that the main 2019-2020 pandemic-shock findings and the 2019-2021 non-reversion patterns are not driven by the most over- or under-represented CBGs in the mobility panel. Detailed tables for this sensitivity analysis are reported in the Supplementary material.

#### 5.3.9 Spatial autocorrelation diagnostics

Since the analysis is conducted at the CBG level and the socioeconomic clusters exhibit spatial structure, we also examined spatial autocorrelation in CBG-level parameter changes. Specifically, we computed global Moran's *I* for the changes in each calibrated parameter across 2018–2019, 2019–2020, and 2020–2021. Because Queen contiguity produced a small number of isolated CBGs, we used five-nearest-neighbor spatial weights. The results show that pre-pandemic 2018–2019 changes exhibit little evidence of spatial autocorrelation. In contrast, several pandemic-era changes from 2019 to 2020 and from 2020 to 2021 show statistically significant but modest spatial autocorrelation.

For the 2019–2020 changes, the global Moran's *I* values are 0.157 for store area, 0.136 for chain loyalty, 0.101 for POI count, 0.073 for POI diversity, 0.006 for demographic similarity, and 0.105 for CBG-store distance. For the 2020–2021 changes, the corresponding values are 0.104 for store area, 0.063 for chain loyalty, 0.046 for POI count, 0.037 for POI diversity, 0.002 for demographic similarity, and 0.093 for CBG-store distance. These diagnostics suggest that pandemic-era shifts in revealed shopping-location patterns had a spatial component. However, the magnitudes of the global Moran's *I* values indicate that the main findings are not solely driven by strong spatial clustering. Local indicators of spatial association could be examined in future research to identify local areas of spatial clustering in parameter changes.

#### 5.3.10 Estimating the impact of the pandemic on revealed store-selection shifts

The rise of the COVID-19 pandemic in early 2020 as an exogenous shock, along with access to historical visits data, enables us to investigate the impact of the pandemic on changes in revealed shopping-location patterns in a quasi-experimental setting. Using a difference-in-differences (DID) method, we compare changes in calibrated parameters from 2018 to 2019 (control group) with changes from 2019 to 2020 (treatment group), treating the 2019–2020 pandemic-shock period as the treatment period and 2018–2019 as the pre-shock comparison period. For comparison, we implemented DID on the entire NYC population, and also the subgroups defined by high-concentration socioeconomic groups and unsupervised clustering tools.

Figure 14 displays the coefficients obtained from the DID tests. Notably, significant changes are observed in the revealed weights associated with store area and chain loyalty, while distance shows an opposite trend. These shifts are particularly prominent among the seven communities identified based on each individual socioeconomic factor. A noteworthy observation is the significant increase in the importance of chain loyalty compared to other parameters among the top 5% groups with the highest proportion of Asian and Hispanic populations. This trend indicates a stronger concentration of visits in specific store chains among these communities, while exhibiting less exploratory behavior.

**Figure 14:**
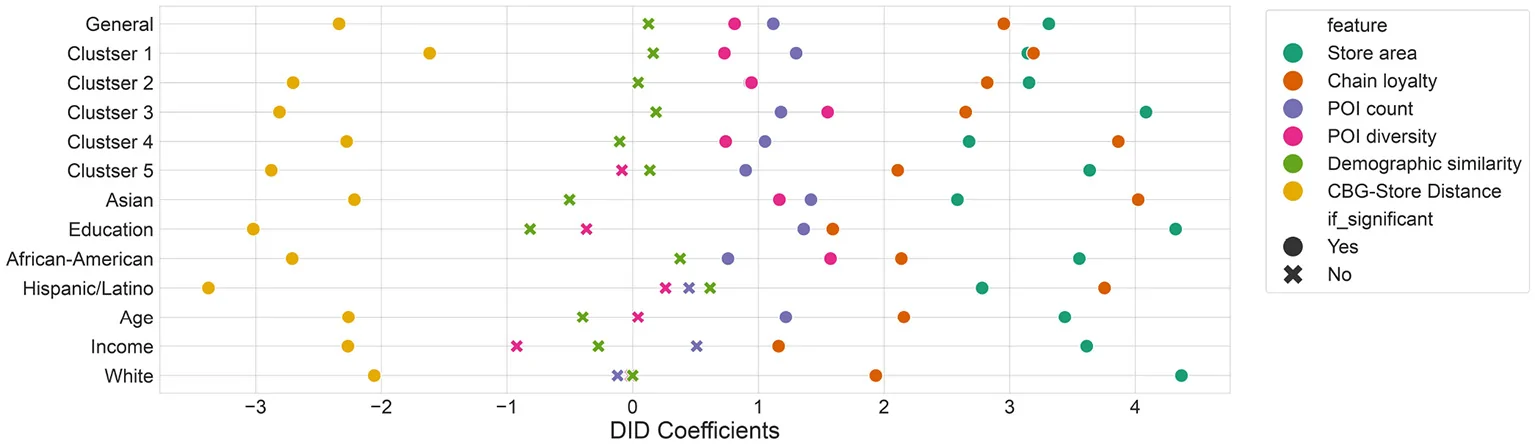
DID analysis results for calibrated parameter value changes. In this figure, the *x* axis shows the range of DID coefficients obtained from the comparison between the calibrated parameter value changes from 2018 to 2019 and from 2019 to 2020. The *y* axis lists different communities that we specifically investigated the impact of COVID-19, including the whole population of NYC residents, the five clusters identified by K-means clustering, and the top 5% society in terms of each single socioeconomic factor. As the legend box shows, the significant coefficients at the two-sided 5% level are presented as dots, while the insignificant ones are visualized by the **×** marks.

For the general population and all communities, there was a noticeable increase in the revealed weights associated with chain loyalty and the count of POIs in the selection of general merchandise and department stores during the pandemic. Specifically, among the top 5% of the White population, there was a stronger inclination toward chain loyalty, with less emphasis on the surrounding POI counts and diversity. Moreover, the DID analysis results suggest that the location and physical characteristics of the store itself had a greater impact on attracting customers than the attractiveness of its nearby commercial or leisure districts.

Furthermore, the analysis of the DID coefficients reveals that all groups examined in the comparison maintained a relatively consistent weight associated with demographic similarity. However, there were variations in the significance levels among communities when it came to the POI aspects. Finally, regardless of the communities, it is evident that the overall ranking of the parameters among the significant coefficients is as follows: store area, chain loyalty, POI features, and CBG-store distance. These findings highlight the importance of store size, prior chain visitation, and the surrounding POI environment in reshaping revealed store-selection patterns during the COVID-19 pandemic.

## 6 Discussion and conclusion

Our study introduces a new approach to measure and quantify changing revealed store-selection patterns for in-person department-store shopping over time. We employ a modified version of the Huff gravity model, incorporating its MCI extension, which allows for the simultaneous consideration of multiple decision-making factors. By leveraging this modified model, our proposed method effectively captures the dynamic nature of shopping-location choices as observed through mobility flows. It achieves this by analyzing the intricate interplay between factors such as customer socioeconomic status, store attributes, surrounding neighborhood characteristics, customer-store proximity, and prior revealed chain visitation.

The findings of our study reveal that the residents of NYC made substantial adjustments in their selection of general merchandise and department stores in response to the COVID-19 pandemic. The quasi-experimental comparison indicates that these changes were associated with the pandemic period. Furthermore, during the early recovery and reopening period in 2021, various governmental non-pharmaceutical and pharmaceutical interventions, including vaccination and relaxation of restrictions, continued to influence these transformations. It is important to note that we do not interpret 2021 as a fully post-pandemic equilibrium. Rather, within the available 2018–2021 observation window, the results indicate that revealed shopping-location patterns did not fully return to their 2019 pre-pandemic baseline by 2021.

We further examined the heterogeneity in revealed store-selection shifts among different communities. Initially, we analyzed the behavioral characteristics of CBGs that ranked in the top 5% for each individual socioeconomic attribute. The distinct dissimilarities observed among these high-concentration groups prompted us to employ an unsupervised clustering technique, specifically K-means clustering, to identify different clusters within NYC using all the socioeconomic features combined. To address the sensitivity of the top 5% threshold, we also examined top 10% groups and conducted PCA-based socioeconomic structure checks. These additional analyses support the robustness of the main heterogeneity patterns and indicate that the results are not driven only by a single threshold-based grouping rule.

Regardless of whether they belonged to the top 5% single demographic groups or the clusters identified by K-means, consumers across these clusters exhibited a similar shift in their revealed store-selection weights from 2019 to 2020. They placed a higher revealed weight on store size, as well as one or more factors among chain loyalty, POI count, and POI diversity, while their revealed weight associated with customer-store distance decreased. This trend aligns with the observed broader trend at the population level. As society began to recover from the outbreak in 2021, the occupants of these clusters continued to place lower weight on distance when selecting general merchandise and department stores. However, their calibrated weights for other aspects experienced a variety of changes. The magnitudes of these shifts from 2020 to 2021 were notably smaller compared to the changes observed from 2019 to 2020, suggesting partial adjustment rather than full reversion to the pre-pandemic baseline.

Motivated by the aforementioned findings, we employed the difference-in-differences (DID) technique to estimate the impact of the pandemic on the temporal shifts in calibrated parameters across the entire population and various subgroups. Through our analysis, we confirmed that chain loyalty exhibited the strongest positive shift among CBGs with a high Asian resident proportion. Moreover, the outlook toward each parameter varied among different communities to different degrees, except for demographic similarity, which showed relatively limited changes. Nevertheless, when evaluating the seven demographic groups separately, we consistently observed the patterns above.

Our DID analysis further revealed that the most substantial changes induced by the pandemic, across different clusters, population subgroups, and the overall population of NYC, were evident in the calibrated weights associated with store area and chain loyalty. Remarkably, the shift in the parameter associated with customer-store distance stood out as a noteworthy finding. While being significant and robust, it displayed a reversed trend compared to the other parameters examined. This indicates that the pandemic period was associated with a lower revealed importance of distance, while store scale, prior chain visitation, and the surrounding POI environment gained greater importance in observed shopping mobility patterns.

A fit-stratified sensitivity analysis shows that the main pandemic-shock and non-reversion findings persist after excluding the CBGs with the lowest calibration fit in 2020 and 2021, and remain directionally consistent across all quartiles of the 2021 fit distribution, indicating that these findings are not primarily an artifact of the years in which CBG-level model fit is weakest. A format-level sensitivity analysis further shows that the direction of the store area, POI count, and CBG-store distance shifts is consistent across the Department Store and General Merchandise/Warehouse Club subcategories, while the chain-loyalty increase is disproportionately concentrated among Department Stores in the acute 2020 shock year, a pattern examined directly with a lagged specification of chain loyalty. A PSO estimator-stability analysis confirms that the direction of the main effects is robust across ten independent calibration seeds, although the precise magnitudes carry meaningful seed-to-seed variability. The additional robustness checks strengthen the interpretation of these results. The sampling-bias sensitivity analysis shows that the main 2019–2020 shifts and the 2019–2021 non-reversion patterns remain stable after excluding CBGs with the highest absolute sampling bias values. The K-means stability check shows that the five-cluster socioeconomic partition is not sensitive to random initialization. The spatial autocorrelation diagnostics show that pandemic-era parameter changes had a statistically significant but modest spatial component. Therefore, although neighborhood spillovers and spatial dependence may be present, the magnitudes of the Moran's *I* values suggest that the main findings are not solely driven by strong spatial clustering.

The findings of this study have important implications for retail managers, marketers, and urban planners in adapting to crisis and early recovery conditions. The analysis of revealed store-selection shifts provides valuable insights that can guide decision-making and strategic planning. For retailers, the results suggest that store scale, chain familiarity, and the surrounding amenity environment may become more important under conditions of health risk and mobility uncertainty. For urban planners, the results highlight that crisis-induced changes in shopping mobility may have heterogeneous effects across socioeconomic communities, potentially reshaping access to retail opportunities and everyday services.

In summary, our study results underscore the importance of understanding changing urban shopping mobility patterns to enable businesses and policymakers to effectively adapt their strategies and offerings to meet evolving consumer needs during and after major disruptions. The results suggest that the COVID-19 shock changed revealed shopping-location patterns in NYC and that these patterns did not fully revert to their 2019 baseline by the early recovery period in 2021.

This study represents the first attempt, to the best of our knowledge, to quantify temporal shifts in revealed shopping-location patterns when selecting a retail location for shopping using a modified MCI/Huff gravity framework and large-scale mobility data. The findings provide a potential avenue for future research to further investigate and quantify these shifts in customer retail location decisions. Our proposed approach in utilizing a modified and enhanced version of the MCI variation of the Huff gravity model, combined with quantitative methods and optimization techniques, maximizes the model's fit to the large-scale historical records of customer mobility and visit patterns.

However, it is essential to acknowledge that due to the limited availability of data, we could only incorporate six attractiveness factors out of the various factors introduced in the literature into our study. While the model still yields more informative results compared to unidimensional statistics, it presents limitations in capturing the complete picture. Future research could explore alternative proxies for store attractiveness that have the potential to enhance the model's performance. Additionally, investigating different approaches for determining the optimal parameters of the model could be a worthwhile pursuit. Determining the most suitable factors to incorporate and identifying readily available ones present opportunities for further investigation and future research. Moreover, the possibility of replacing the current proxies for store attractiveness, based on the count and diversity of POIs in the store's vicinity, with more comprehensive factors derived from assessing retail agglomerations, warrants further investigation.

While the mobility dataset utilized in this study provides a valuable and large-scale view of urban shopping mobility, it is important to acknowledge that mobile-location data may contain sampling biases. The observed population is limited to smartphone users who use applications included in the data panel and have enabled location tracking. Although our representativeness diagnostics and sampling-bias sensitivity analysis suggest that the main findings are stable, these tests cannot eliminate all possible sources of sampling bias. Therefore, the results should be interpreted as patterns inferred from the observed mobility panel rather than as perfect population-level measurements without uncertainty.

A related limitation concerns the decline in average model fit during 2020 and 2021 relative to the pre-pandemic years, discussed in Section 5 and examined directly in the fit-stratified sensitivity analysis reported in Section 5.3.4. Although that analysis indicates that the direction of the main pandemic-shock and non-reversion findings is not driven by the subset of CBGs with the weakest calibration fit, the magnitude of some parameter estimates, particularly POI count, is more sensitive to CBG-level fit than others. Given the lower and more variable fit in 2020 and 2021, the precise magnitudes of the calibrated parameters in these years should be interpreted with somewhat more caution than the pre-pandemic estimates, even though the qualitative pattern of increased weight on store area and chain loyalty and decreased weight on distance appears robust to this concern.

Another important limitation is related to the study period. The available mobility panel covers the years 2018 to 2021. Therefore, 2021 is interpreted as an early recovery and reopening period, rather than as a fully post-pandemic period. As a result, this study cannot determine whether the observed changes persisted into a later stabilized post-pandemic equilibrium. Future studies with data from 2022 and later years could investigate whether the revealed shopping-location patterns identified in this paper continued, reversed, or evolved into new forms.

The interpretation of the results is also limited by the fact that observed shopping flows can be shaped by both demand-side and supply-side conditions. All stores included in the analysis were confirmed to be active in the dataset across the study period, and there were not enough confirmed closure cases in the analytical store set to support a formal closure analysis. Nevertheless, temporary changes in operating hours, capacity restrictions, service availability, and other pandemic-related operational constraints could still influence observed visits. Therefore, the calibrated parameters should be interpreted as revealed store-selection weights under the combined demand-side and supply-side conditions present during each year, rather than as direct measures of pure consumer preferences.

Our study is further limited by the absence of data on spending and transactions across various sales channels. This limitation impedes our ability to establish any association between the utilization of different purchase channels and store location choice. Consequently, future research should prioritize investigating the relationship between the utilization of diverse sales channels and the decision-making process regarding store location. Another important area for exploration would be the analysis of the heterogeneity in utilization of different purchase channels among different socioeconomic groups, along with an examination of the underlying dynamics that drive these patterns.

Finally, future research could extend this work by examining retail mobility as a network of origin-destination connections between residential neighborhoods and store locations or retail districts. Such an approach could investigate which neighborhood-to-retail corridors remained stable during the COVID-19 shock and which became more fragile, building on mobility-network approaches to pandemic inequality and reopening dynamics (Chang et al., 2021). Similarly, perturbation-based stability analysis could be used to evaluate whether fitted store-selection patterns are robust to changes in the mobility matrix or parameter vectors, which is consistent with recent data-driven and machine-learning analyses of COVID-related mobility and migration changes (Mukhamedova and Tyukin, 2024). These extensions would provide further insight into the resilience of urban retail mobility systems under major disruptions.

## Data Availability

The original contributions presented in the study are included in the article/Supplementary material, further inquiries can be directed to the corresponding author.
