# Supplementary material for: From crisis to new routine: shifts in urban shopping mobility and socioeconomic inequality during and after the COVID-19 shock
Source: Front Big Data. 2026 Sep 15;9:1776172. doi: 10.3389/fdata.2026.1776172 (PMC13619519; doi:10.3389/fdata.2026.1776172)
Supplement: Supplementary file 1 [file Data_Sheet_1.pdf]

Supplementary Material for From Crisis to New Routine: Shifts in Urban Shopping Mobility and Socioeconomic Inequality During and After the COVID-19 Shock

Yilun Xu, Mohsen Bahrami, and Alex Pentland

1 MOBILITY DATA REPRESENTATIVENESS ASSESSMENT

Table S1 displays the Pearson correlation coefficients (r) between the tracked device counts in the mobility data panel and the population size across various geographic, socioeconomic, and demographic levels. The table also provides additional information, such as the number of observations or groups available for each aggregation. Furthermore, it includes the sum of absolute sampling bias (sum(AB)), median absolute sampling bias (median(AB)), median sampling bias (median(B)), and standard deviation of sampling bias values (sd(B)) for each aggregation, offering further insights into the representativeness of data considering the magnitude and variability of the sampling bias.

The final row of Table S1 presents information about the dataset utilized in the case study of this research. The results suggest that the preprocessed data retains a meaningful relationship between tracked device counts and population size. However, the representativeness of mobile-location data is not uniform across all spatial scales and socioeconomic groups. Therefore, the data should not be interpreted as a perfect random sample of the entire population. Rather, the table provides a descriptive assessment of the observed mobility panel and motivates the additional sampling-bias sensitivity analysis reported in Section 9.

Table S1. Basic statistics of mobility data sampling ratio and bias at different geographic, and socioeconomic levels.

Aggregation	Observations	r	sum(AB)	median(AB)	median(B)	sd(B)
Race	7	1.000	2.926	0.1833	-0.0793	0.6486
Household income	16	0.971	7.35	0.4298	-0.1607	0.5627
Educational attainment	24	0.998	5.524	0.1379	-0.0559	0.4112
State	29	0.994	11.297	0.1778	-0.0421	0.6506
County	1,176	0.976	18.108	0.0042	-0.0019	0.0580
CBG	61,437	0.463	45.192	0.0004	-0.0002	0.0025
CBGs preprocessed	5,502	0.756	24.291	0.0035	-0.0029	0.0046

Note: All required data and code for attaining the results shown in this table are available in the repository of this research project (Section 12).

In addition to the representativeness statistics reported in Table S1, we conducted an additional filtering and representativeness assessment for NYC CBGs. From all 6,493 NYC CBGs, 6,186 residential CBGs remained after applying minimum population and device-count filters. These retained CBGs account for 99.84% of the population and 97.26% of the tracked SafeGraph devices.

The 6,186-CBG sample used in this additional descriptive assessment applies only the minimum population and device-count filters, whereas the final 5,502-CBG analytical sample additionally requires

complete longitudinal visit and demographic records and applies the IQR-based sampling-ratio and sampling-bias filters described in the main manuscript.

Borough-level correlation between census population and SafeGraph device counts remained strong after filtering. However, CBG-level representativeness remained more uneven. This result is consistent with the broader concern that mobile-location datasets may be more reliable at aggregate levels than at the most granular spatial scale.

We also examined representativeness across socioeconomic and demographic groups. After filtering, education representation ratios ranged from approximately 0.900 to 1.062, income representation ratios ranged from approximately 0.888 to 1.055, and race representation ratios ranged from approximately 0.943 to 1.167. These values indicate that the panel is reasonably balanced across several socioeconomic dimensions, while still showing some under- and over-representation for specific groups. For example, the highest-income group was somewhat underrepresented, while some racial groups showed modest over- or under-representation. Therefore, the main manuscript interprets the results as patterns inferred from the observed mobility panel, and not as complete population-level measurements without uncertainty.

2 ANALYSIS OF CHANGE IN DIRECTION OF CALIBRATED PARAMETER SHIFTS

2.1 Top 5% socioeconomic communities

In order to gain deeper insights into the direction of temporal shifts in revealed store-selection patterns, we analyze the change in changes of calibrated parameter values. This change is represented by $\Delta\omega_t$, where ω denotes a calibrated parameter and t represents a specific year. The calculation of $\Delta\omega_t$ is illustrated in Equation S1, which provides a quantitative measure for assessing the magnitude and direction of parameter changes over a two-year period.

$$\Delta\omega_t = (\omega_t - \omega_{t-1}) - (\omega_{t-1} - \omega_{t-2}) \tag{S1}$$

Figures S1 and S2 illustrate the average values of $\Delta\omega_t$ for the top 5% distinctive communities in each of seven single socioeconomic features, in comparison to the population average. Details regarding the definitions of these distinctive communities can be found in Section 5.2 of the main manuscript. $\Delta\omega_t$ values are reported for 2020 and 2021 separately. Since there are no significant shifts in calibrated parameters from 2018 to 2019, the $\Delta\omega_t$ values for 2020 are very similar to the change in parameter values from 2019 to 2020, which makes Figure 6 in the main manuscript and Figure S1 here convey a similar message. Therefore, in the following, we compare the differences in temporal store-selection patterns between communities and the general NYC population from the perspective of each attractiveness factor with a greater focus on values of $\Delta\omega_t$ for 2021, as shown in Figure S2.

Store area: The community with the top 5% Asian resident proportion showed limited change in the revealed weight associated with store area during both 2020 and 2021, indicating stronger concentration in their previously selected chains throughout the pandemic and early recovery period. In contrast, other communities, including those with higher education levels, median incomes, and older age groups, displayed an approximately 20% increased emphasis on store area during the pandemic. This suggests the presence of homogeneous subgroups with socioeconomic features where residents placed a higher revealed weight on store area amid the COVID-19 shock. Interestingly, in 2021, Asian resident CBGs showed relatively little additional change in the weight associated with store area compared to

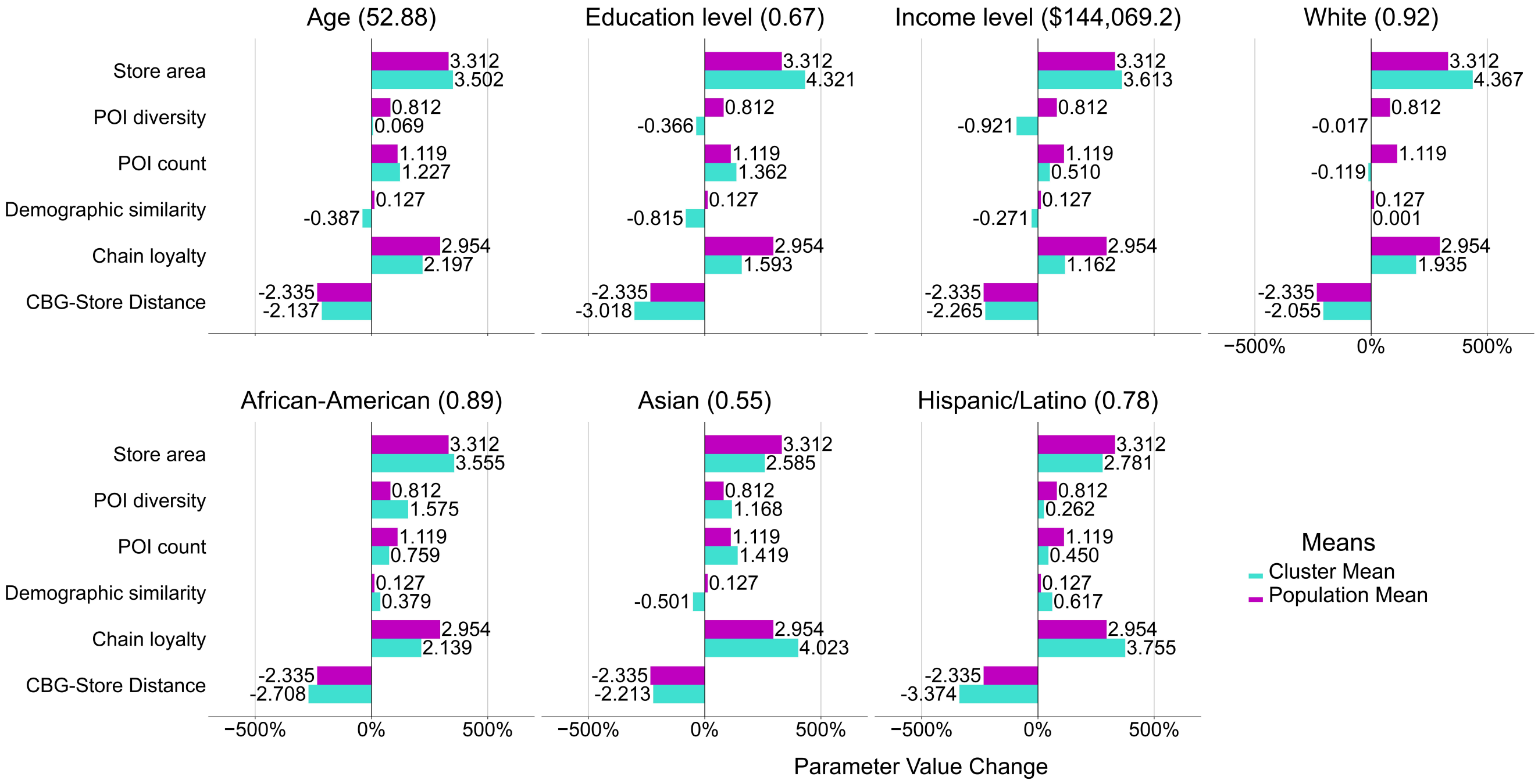

Figure S1. Mean $\Delta\omega_t$ value comparisons in top 5% socioeconomic communities and population as of 2020. This figure shows the average change in changes of calibrated parameter values ($\Delta\omega_t$) for the top 5% Socioeconomic Communities in each sub-figure. The community $\Delta\omega_t$ values are shown by blue bars and for comparison purposes the population level $\Delta\omega_t$ values are shown by purple bars. $\Delta\omega_t$ values are computed using Equation S1.

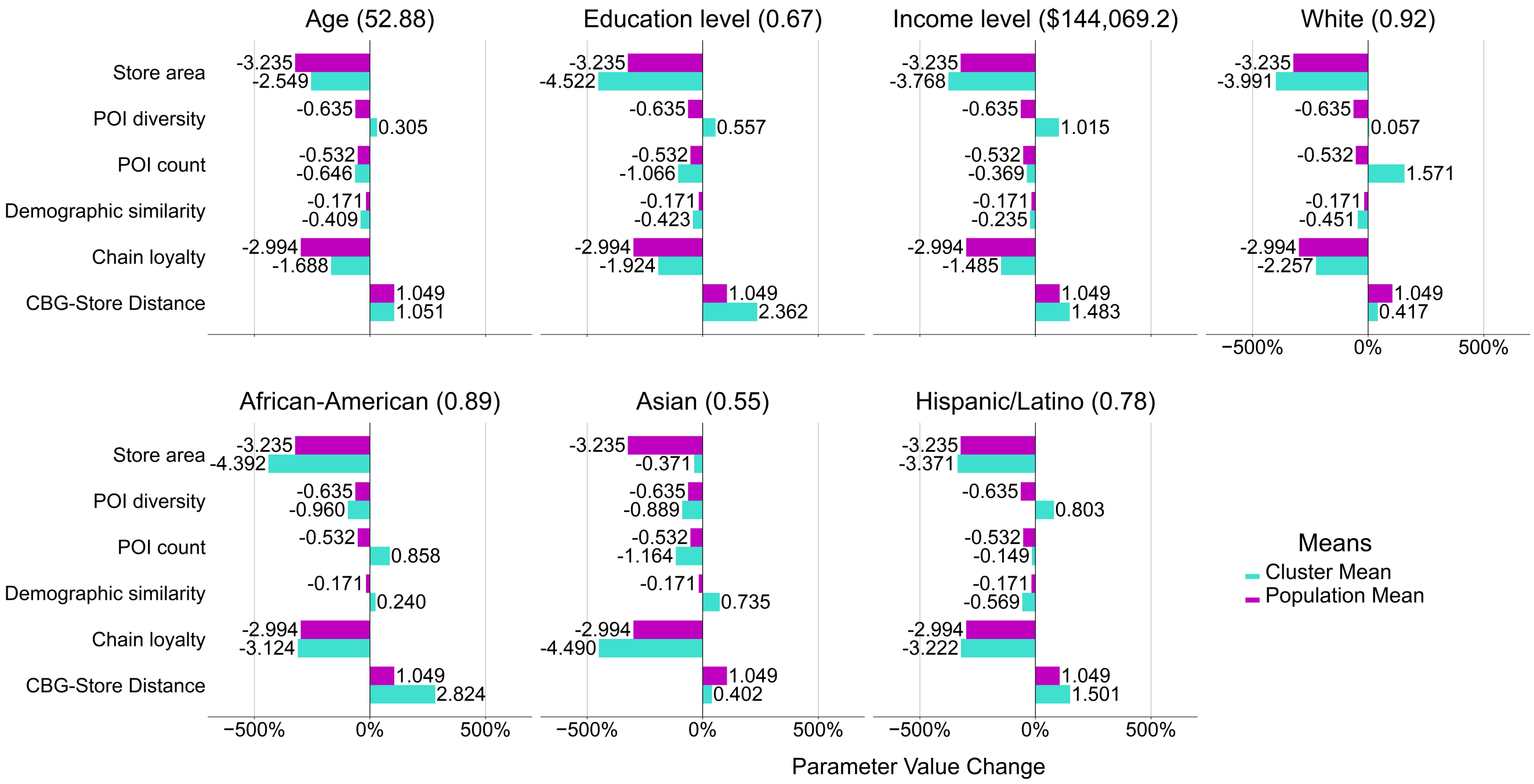

Figure S2. Mean $\Delta\omega_t$ value comparisons in top 5% socioeconomic communities and population as of 2021.

other communities, while communities with higher education levels and median incomes still showed a 20% increase in attention to store area. Additionally, CBGs with residents in the top 5% based on

median age exhibited a significant 15% increase in attention to this feature, even greater than the initial increase when the COVID-19 pandemic started to spread. Conversely, African-American resident CBGs demonstrated a higher revealed weight for store area in their department store selection after the introduction of vaccination and relaxation of social restrictions.

Chain loyalty: The analysis reveals a notable trend among Asian resident CBGs, as their revealed weight associated with chain loyalty experienced a significant increase during and after the pandemic shock. On the other hand, higher income CBGs displayed a relatively lower level of change in chain loyalty, suggesting a greater degree of indifference towards this factor in their revealed store-selection patterns.

POI count: During the COVID-19 pandemic, CBGs with higher levels of education and African-American resident CBGs showed an increased revealed weight associated with the abundance of POIs in department store selection. However, in 2021, CBGs with a higher proportion of Asian residents, higher levels of education, and a higher median age among their residents showed a higher revealed weight for stores that had a greater number of POIs in close proximity.

POI diversity: During the pandemic and as society began to recover in 2021, African-American resident CBGs emerged as the leading group in placing a strong revealed weight on the diversity and richness of POIs. This indicates stronger observed selection of retail locations that offer a wider range of nearby amenities and services.

Demographic similarity: Residents residing in CBGs with higher levels of education and income displayed a stronger tendency to select stores located in neighborhoods that shared greater demographic similarities with their own community. This observation suggests that the store-selection process may be partly shaped by the socioeconomic characteristics of the communities to which residents belong.

CBG-Store distance: As society transitioned into the early recovery period, CBGs characterized by higher education levels, a larger African-American population, and an older population demonstrated a greater increase in their revealed weight associated with this factor compared to the general population.

2.2 Clusters by combined socioeconomic characteristics: Five K-means clusters

This section aims to analyze and compare the temporal store-selection patterns among five clusters that have been defined using the K-means algorithm and are based on combined socioeconomic characteristics. We also include the general NYC population for comparison. Specifically, we examine the differences in calibrated parameter changes for each attractiveness factor and place a particular emphasis on understanding the direction of change over time. Details regarding the definitions of these distinctive communities can be found in the main manuscript.

Figures S3 and S4 show changes in the value of $\Delta\omega_t$ between 2020 and 2021. From 2020 to 2021, $\Delta\omega_t$ changes reversed roughly among each community and the entire population. The earlier increase in the revealed weights associated with store size, chain loyalty, POI count, and POI diversity from 2019 to 2020 began to decline from 2020 to 2021, while the reduced role of CBG-store distance persisted in 2021. The magnitudes of the changes in 2021, nonetheless, are not as large as those in 2020. This occurrence confirms our earlier observation that following the outbreak, the revealed store-selection patterns of NYC residents changed from their pre-pandemic behavior and the impact of COVID-19 had not fully disappeared by the early recovery period in 2021.

It has also been observed that changes in $\Delta\omega_t$ due to store area, chain loyalty and CBG-store distance are apparently greater than those in POI count, POI diversity and demographic similarity in 2020, while in 2021

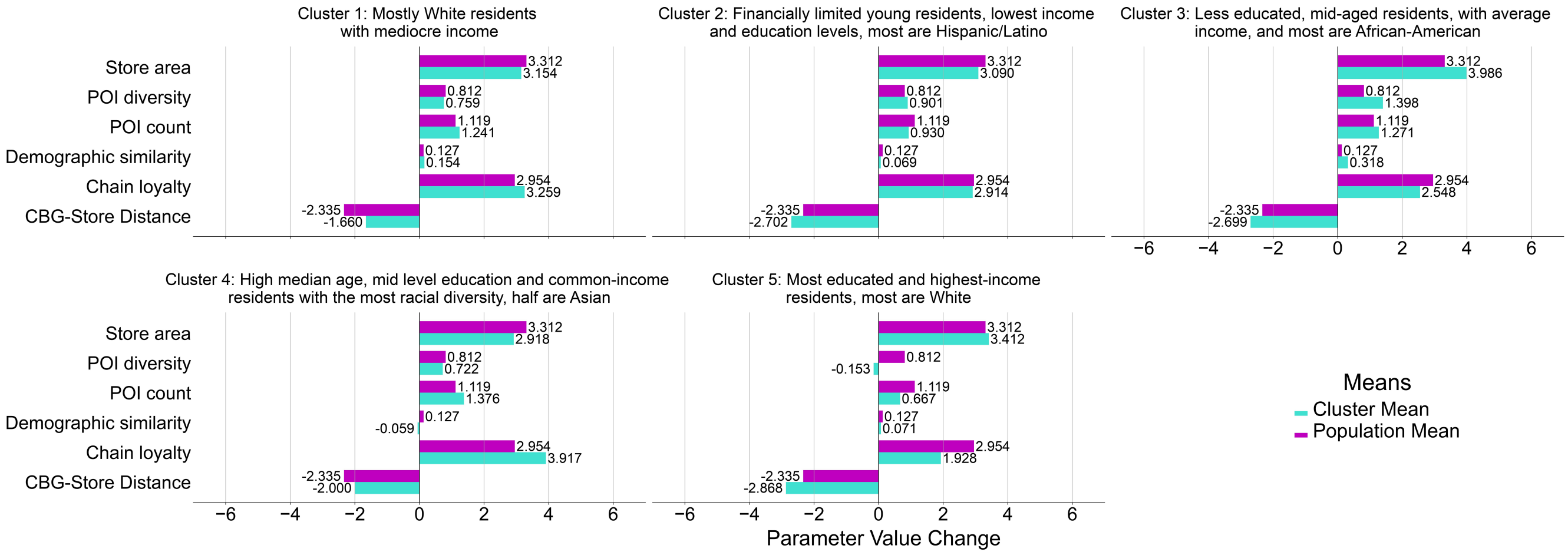

Figure S3. Mean $\Delta\omega_t$ value comparisons in clusters by K-means as of 2020.

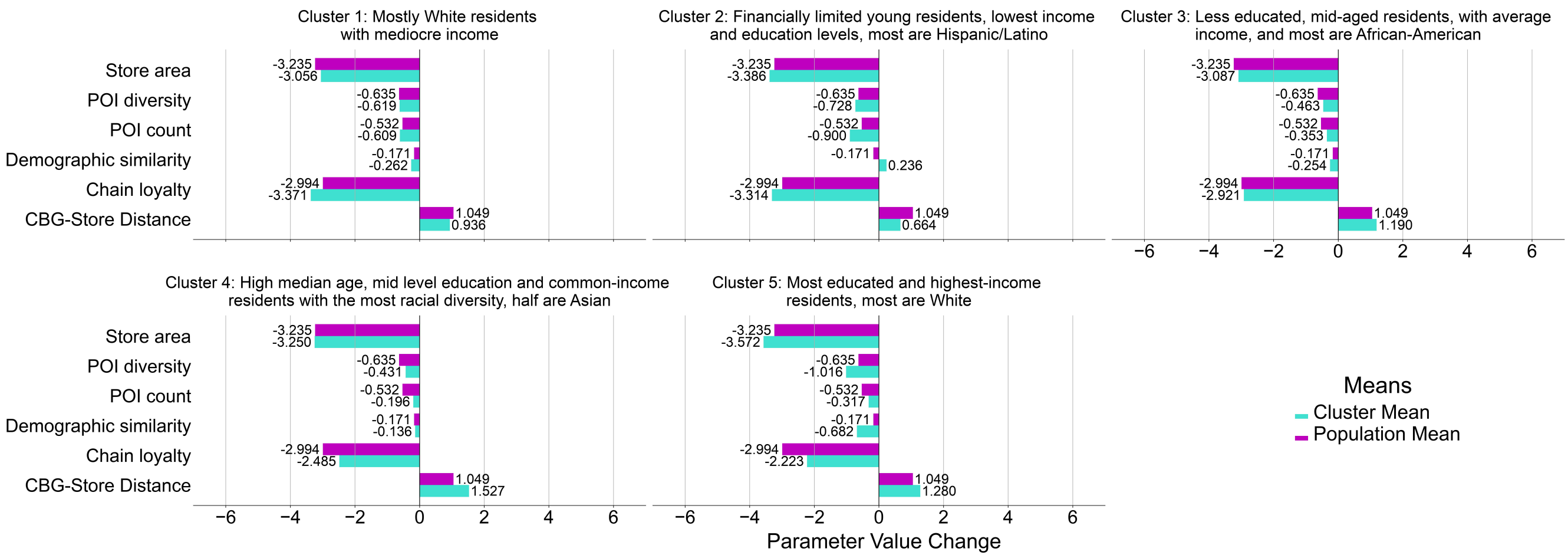

Figure S4. Mean $\Delta\omega_t$ value comparisons in clusters by K-means as of 2021.

changes in $\Delta\omega_t$ in CBG-store distance are similar to those of POI count and diversity. Consequently, it has been signaled that since the outbreak of COVID-19, the characteristics of stores and revealed prior chain visitation gained more importance relative to nearby commercial services and commuting cost. Overall, our findings using unsupervised clustering confirm that the pandemic response was heterogeneous among different communities.

3 ROBUSTNESS OF SOCIOECONOMIC GROUP DEFINITIONS

3.1 Top 10% socioeconomic communities

The main manuscript uses top 5% socioeconomic and demographic groups to identify high-concentration communities with distinctive characteristics. Since any fixed threshold may be considered somewhat arbitrary, we repeated the analysis using top 10% groups. The threshold values for defining these groups are reported in Table S2. As expected, the top 10% groups are broader and less extreme than the top 5% groups.

Table S2. Comparison of top 5% and top 10% thresholds for socioeconomic and demographic groups.

Socioeconomic attribute	Top 5% threshold	Top 10% threshold
Education level	0.67	0.57
Median age	52.88	48.86
Median income	$144,069.2	$115,443.4
White resident proportion	0.92	0.87
African-American resident proportion	0.89	0.78
Asian resident proportion	0.55	0.41
Hispanic/Latino resident proportion	0.78	0.68

Note: The values show the minimum value required for a CBG to be included in the corresponding high-concentration group.

The top 10% results are consistent with the main findings. For the 2019-2020 pandemic-shock comparison, the revealed weight associated with CBG-store distance decreases across all seven top-decile groups, while the revealed weights associated with store area and chain loyalty increase across all seven top-decile groups. POI count also generally increases, while POI diversity and demographic similarity show weaker and more mixed patterns. For the 2020-2021 comparison, the results suggest partial adjustment rather than full reversion to the 2019 baseline. These findings indicate that the main socioeconomic heterogeneity results are not driven only by the top 5% threshold.

3.2 PCA-based socioeconomic structure check

To further examine whether the observed heterogeneity is consistent with broader multivariate socioeconomic structure, we conducted a principal component analysis (PCA) using the socioeconomic and demographic attributes of NYC CBGs. Table S3 reports the explained variance of the first five principal components.

Table S3. Explained variance of the first five principal components.

Component	Explained variance	Cumulative variance
PC1	42.10%	42.10%
PC2	18.37%	60.47%
PC3	16.93%	77.40%
PC4	11.14%	88.55%
PC5	7.06%	95.61%

The first three principal components explain 77.4% of the variation, while the first four explain 88.5%. The first component broadly captures an advantage or affluence dimension, with positive loadings on education, income, and White resident share, and negative loadings on Black and Hispanic/Latino resident shares. The second component is strongly related to Black resident share, while the third component is strongly related to Hispanic/Latino resident share and age. The fourth component is strongly related to Asian resident share. These results provide additional support that the heterogeneity observed in the main analysis is related to broader socioeconomic structure and is not only an artifact of a single threshold-based grouping rule.

4 K-MEANS CLUSTERING STABILITY CHECK

To assess whether the five-cluster socioeconomic partition is sensitive to random initialization, we reran the K-means procedure using standardized socioeconomic and demographic variables under 10 random seeds. We then compared the resulting partitions using pairwise Adjusted Rand Index (ARI) values. The results are summarized in Table S4.

Table S4. K-means clustering stability results across random seeds.

Metric	Value
Number of random seeds	10
Number of clusters	5
Minimum pairwise ARI	0.995308
Mean pairwise ARI	0.998759
Median pairwise ARI	1.000000
Maximum pairwise ARI	1.000000
Mean inertia	15175.504
Standard deviation of inertia	0.00745
Best seed by lowest inertia	12424

The ARI values range from 0.995 to 1.000, with a mean value of 0.999 and a median value of 1.000. The inertia values are also highly stable across random seeds. These results indicate that the five-cluster solution is not sensitive to random initialization and that the cluster-level heterogeneity results are not driven by a particular K-means starting condition.

The cluster sizes in the final solution are reported in Table S5.

Table S5. Cluster sizes in the K-means stability run.

Cluster	Size	Interpretation from centers
Cluster 1	1,585	Predominantly White, moderate-income, moderate education
Cluster 2	1,387	Lower-income, younger, majority Hispanic/Latino
Cluster 3	1,198	Majority African-American, moderate/lower income
Cluster 4	811	Higher Asian share, moderate income, older/racially diverse
Cluster 5	840	Highest income, highest education, predominantly White

5 FIT-STRATIFIED SENSITIVITY ANALYSIS

This section reports full results for the fit-stratified sensitivity analysis summarized in the main manuscript. The main text reports the 2019 to 2020 and 2019 to 2021 comparisons after excluding CBGs with the lowest model fit in the relevant post-shock year; Table S6 extends this to the 2020 to 2021 comparison.

As a more stringent test, we split CBGs into quartiles of model fit and repeat the main comparisons within each quartile. Table S7 reports the 2019 to 2020 comparison by quartile of 2020 fit, and Table S8 reports the 2019 to 2021 comparison by quartile of 2021 fit.

Table S6. Fit-stratified sensitivity of calibrated parameter changes from 2020 to 2021.

Parameter	Full sample	Excl. bottom 10% fit	Excl. bottom 20% fit	Excl. bottom 25% fit
Store area	+1.34%	+0.71%	+1.84%	+4.69%
Chain loyalty	+0.26%	-1.20%	-0.58%	-0.36%
POI count	+13.15%	+4.78%	+0.39%	+3.63%
POI diversity	+3.23%	+2.58%	+2.39%	+3.21%
Demographic similarity	-0.32%	-0.73%	-1.18%	-1.05%
CBG-store distance	-15.97%	-16.20%	-16.93%	-15.09%

Note: CBGs are excluded based on their 2021 model fit (one minus PSO calibration cost).

Table S7. Percentage change in calibrated parameter values from 2019 to 2020, by quartile of 2020 CBG-level model fit.

Parameter	Q1 (lowest fit)	Q2	Q3	Q4 (highest fit)
Store area	+65.99%	+79.67%	+87.03%	+81.15%
Chain loyalty	+41.40%	+54.87%	+41.56%	+36.80%
POI count	+42.13%	+29.98%	+19.57%	+19.03%
POI diversity	+10.34%	+10.99%	+13.26%	+14.22%
Demographic similarity	+4.23%	-0.15%	+2.69%	+3.26%
CBG-store distance	-36.77%	-26.86%	-21.12%	-5.39%

Note: Quartiles (n≈1,376 CBGs each) are defined on the distribution of 2020 CBG-level model fit.

Table S8. Percentage change in calibrated parameter values from 2019 to 2021, by quartile of 2021 CBG-level model fit.

Parameter	Q1 (lowest fit)	Q2	Q3	Q4 (highest fit)
Store area	+62.09%	+67.50%	+99.87%	+95.19%
Chain loyalty	+36.99%	+42.30%	+55.54%	+42.05%
POI count	+92.23%	+9.31%	+38.05%	+40.76%
POI diversity	+14.84%	+13.06%	+17.54%	+17.89%
Demographic similarity	+4.49%	+2.51%	+2.03%	-0.27%
CBG-store distance	-37.61%	-42.24%	-31.67%	-28.16%

Note: Quartiles (n≈1,376 CBGs each) are defined on the distribution of 2021 CBG-level model fit.

The direction of change for store area, chain loyalty, POI diversity, and CBG-store distance is consistent across all four quartiles in both tables, including the lowest-fit quartile. The magnitude of the CBG-store distance decline is notably larger among lower-fit CBGs in the 2019 to 2020 comparison (-36.77% in the lowest quartile versus -5.39% in the highest quartile), which we interpret as a sign that lower calibration fit may inflate the magnitude, but not the direction, of the estimated distance effect. POI count shows the least consistent pattern across quartiles, consistent with it being the parameter most sensitive to the fit filter in Table 6 of the main manuscript.

6 FORMAT-LEVEL (NAICS) SENSITIVITY ANALYSIS

This section reports full details for the format-level sensitivity analysis summarized in the main manuscript, including the store-category classification procedure, sample construction, and the 2020 to 2021 comparison omitted from the main text for space.

Store categories were assigned using a two-stage validated join against SafeGraph's Places reference data: first by matching each store's `safegraph_place_id` directly (available for 47 of the 282 stores), and second, for the remaining stores, by matching the brand-level identifier of the store's chain. Place-level and brand-level classification agreed on every one of the 18 brands for which both were available, supporting the reliability of the brand-level classification for the remaining stores. Table S9 reports the final classification by brand.

Table S9. Final store-category classification by brand.

Brand	Stores (n)	NAICS code	Category
Dollar Tree	77	452319	General Merchandise
Family Dollar Stores	58	452319	General Merchandise
Dollar General	26	—	Unresolved
T.J. Maxx	22	452210	Department Stores
Target	20	452210	Department Stores
Sears Home Services	12	452210	Department Stores
Five Below	10	452319	General Merchandise
Macy's	9	452210	Department Stores
BJ's Wholesale Club	8	—	Unresolved
Bloomingdale's	6	452210	Department Stores
Barneys New York	5	452210	Department Stores
Costco Wholesale Corp.	5	452319	General Merchandise
Hermès	4	452210	Department Stores
Kohl's	3	—	Unresolved
JCPenney	3	452210	Department Stores
Kmart	3	452319	General Merchandise
Neiman Marcus	2	452210	Department Stores
Saks Off Fifth	2	452210	Department Stores
Sears	2	—	Unresolved
Lou & Grey	1	452210	Department Stores
Saks Fifth Avenue	1	452210	Department Stores
DAISO	1	—	Unresolved
Bottega Veneta	1	452210	Department Stores
Amazon 4-Star	1	452319	General Merchandise

Note: "Unresolved" brands do not appear in either SafeGraph Places reference file available to us and are retained in the pooled analysis reported throughout the paper but excluded from the format-level sensitivity analysis. Totals: 88 Department Stores, 154 General Merchandise Stores, 40 unresolved; 282 stores overall.

For each resolved format, we identified the pool of CBGs with nonzero visits to that format's stores in all four study years, and drew a stratified random sample balanced by borough and household-income quintile (Table S10). Sample percentages matched pool percentages within approximately 0.5 percentage points on both stratification dimensions for both formats.

Table S10. Eligible CBG pools and stratified sample sizes by format.

Format	Eligible CBGs (visits > 0, all 4 years)	Stratified sample
Department Stores (452210)	4,997	448
General Merchandise (452319)	5,276	450

Note: Samples are stratified by NYC borough and household-income quintile using a fixed random seed.

Table S11 reports the 2020 to 2021 comparison omitted from the main text.

Table S11. Percentage change in calibrated parameter values from 2020 to 2021: pooled sample versus format-restricted subsamples.

Parameter	Pooled (n=5,502)	Dept. Stores 452210 (n=448)	Gen. Merch. 452319 (n=450)
Store area	+1.34%	-2.82%	-2.05%
Chain loyalty	+0.26%	+1.38%	+1.46%
POI count	+13.15%	+22.84%	+30.72%
POI diversity	+3.23%	+1.21%	-12.36%
Demographic similarity	-0.32%	-4.38%	-3.13%
CBG-Store Distance	-15.97%	-9.49%	-16.06%

Table S12 reports mean CBG-level model fit by format and year, confirming that the fit decline documented in the main manuscript is not an artifact of pooling formats.

Table S12. Mean CBG-level model fit by format and year.

Format	2018	2019	2020	2021
Department Stores (452210)	0.713	0.720	0.242	0.210
General Merchandise (452319)	0.704	0.709	0.238	0.171

7 LAGGED CHAIN-LOYALTY SENSITIVITY ANALYSIS

This section reports the mechanical-concentration diagnostics and full lagged-specification results underlying the corresponding robustness check in the main manuscript.

Table S13 reports the CBG-level correlation between changes in raw visit-pattern metrics and changes in brand-level visit concentration (the population-level analog of the chain-loyalty measure), for the pooled sample and each format.

Table S14 reports the full placebo check comparing the true (unlagged) 2019 calibration against a specification that substitutes 2018 loyalty while still fitting the true 2019 visits, for all six parameters and all three samples.

Tables S15 and S16 report the full six-parameter comparison between the same-period (unlagged) and lagged specifications, for the pooled sample and each format. The main manuscript's interpretive focus is

Table S13. Correlation between changes in visit patterns and changes in brand-level concentration.

Sample	Year-pair	corr(Δtotal visits, Δtop-brand share)	corr(Δn chains visited, Δtop-brand share)
Pooled	2019-2020	-0.015	-0.293
Pooled	2019-2021	+0.167	-0.436
Dept. Stores 452210	2019-2020	+0.037	-0.510
Dept. Stores 452210	2019-2021	+0.204	-0.613
Gen. Merch. 452319	2019-2020	+0.032	-0.525
Gen. Merch. 452319	2019-2021	+0.104	-0.567

Note: "Top-brand share" is the largest brand-level visit share within a CBG, computed after aggregating visits to the brand level; it is the population-level analog of the chain-loyalty measure.

Table S14. Placebo check: unlagged versus lagged 2019 calibration (fit to true 2019 visits).

Parameter	Sample	Unlagged 2019	Lagged 2019	% difference
Store area	Pooled	4.2654	4.4474	+4.27%
Chain loyalty	Pooled	6.4996	6.3288	-2.63%
POI count	Pooled	3.8277	3.8520	+0.64%
POI diversity	Pooled	6.7419	6.8306	+1.32%
Demographic similarity	Pooled	7.6131	7.6753	+0.82%
Distance	Pooled	10.6849	10.8295	+1.35%
Store area	Dept. Stores 452210	4.3677	4.1951	-3.95%
Chain loyalty	Dept. Stores 452210	7.1395	6.6635	-6.67%
POI count	Dept. Stores 452210	3.4076	3.5059	+2.88%
POI diversity	Dept. Stores 452210	7.0892	7.3782	+4.08%
Demographic similarity	Dept. Stores 452210	7.8643	7.7572	-1.36%
Distance	Dept. Stores 452210	10.9135	10.8844	-0.27%
Store area	Gen. Merch. 452319	5.6470	5.8258	+3.17%
Chain loyalty	Gen. Merch. 452319	7.7368	7.5598	-2.29%
POI count	Gen. Merch. 452319	3.6785	4.0098	+9.01%
POI diversity	Gen. Merch. 452319	6.5172	6.4790	-0.59%
Demographic similarity	Gen. Merch. 452319	7.4362	7.5062	+0.94%
Distance	Gen. Merch. 452319	10.4610	10.6049	+1.38%

Note: No parameter in any sample exceeds a 15% flag threshold; the largest absolute difference is 9.01%. This confirms the lagging procedure introduces no material distortion in a period already shown to have no significant parameter shift.

on chain loyalty specifically, since that is the parameter under scrutiny; the other parameters are reported here for completeness and are not separately interpreted.

8 PSO ESTIMATOR-STABILITY ANALYSIS

This section reports full results for the PSO estimator-stability analysis summarized in the main manuscript. We first confirmed that our calibration procedure is reproducible: two independent PSO runs on the same CBG under the same fixed random seed produced identical results. We then drew a stratified random sample of 300 CBGs, balanced by borough and household-income quintile (Table S17), and re-calibrated the model for all four years under ten different random seeds (12,000 individual PSO runs).

Table S18 reports the median per-CBG coefficient of variation (standard deviation divided by the absolute mean, computed across the ten seeds for each CBG-year, then the median taken over the 300 CBGs) for each parameter and year. Individual CBG-level estimates carry considerable seed-to-seed noise.

Table S15. Same-period (unlagged) versus lagged percentage change, 2019 to 2020, all parameters.

Parameter	Sample	Unlagged	Lagged
Store area	Pooled	+78.58%	+76.84%
Chain loyalty	Pooled	+43.46%	+24.82%
POI count	Pooled	+27.93%	+41.07%
POI diversity	Pooled	+12.20%	+15.65%
Demographic similarity	Pooled	+2.50%	+3.75%
Distance	Pooled	-22.53%	-18.81%
Store area	Dept. Stores 452210	+58.62%	+98.55%
Chain loyalty	Dept. Stores 452210	+33.85%	+4.10%
POI count	Dept. Stores 452210	+31.77%	+52.72%
POI diversity	Dept. Stores 452210	+8.20%	+4.76%
Demographic similarity	Dept. Stores 452210	-1.51%	+2.44%
Distance	Dept. Stores 452210	-29.06%	-23.66%
Store area	Gen. Merch. 452319	+47.47%	+23.15%
Chain loyalty	Gen. Merch. 452319	+3.41%	-1.68%
POI count	Gen. Merch. 452319	+50.12%	+61.97%
POI diversity	Gen. Merch. 452319	+25.63%	+26.73%
Demographic similarity	Gen. Merch. 452319	+10.25%	-2.33%
Distance	Gen. Merch. 452319	-28.02%	-24.53%

Table S16. Same-period (unlagged) versus lagged percentage change, 2019 to 2021, all parameters.

Parameter	Sample	Unlagged	Lagged
Store area	Pooled	+80.97%	+83.10%
Chain loyalty	Pooled	+43.84%	+40.30%
POI count	Pooled	+44.75%	+65.51%
POI diversity	Pooled	+15.82%	+16.69%
Demographic similarity	Pooled	+2.18%	+3.06%
Distance	Pooled	-34.90%	-27.54%
Store area	Dept. Stores 452210	+54.15%	+107.11%
Chain loyalty	Dept. Stores 452210	+35.70%	+19.93%
POI count	Dept. Stores 452210	+61.87%	+79.70%
POI diversity	Dept. Stores 452210	+9.51%	+11.23%
Demographic similarity	Dept. Stores 452210	-5.82%	+8.51%
Distance	Dept. Stores 452210	-35.80%	-22.63%
Store area	Gen. Merch. 452319	+44.45%	+34.59%
Chain loyalty	Gen. Merch. 452319	+4.91%	-3.04%
POI count	Gen. Merch. 452319	+96.24%	+83.87%
POI diversity	Gen. Merch. 452319	+10.11%	+14.90%
Demographic similarity	Gen. Merch. 452319	+6.81%	+5.56%
Distance	Gen. Merch. 452319	-39.58%	-34.86%

Table S19 reports the mean and minimum pairwise Pearson correlation, across the ten seeds, of each parameter's cross-CBG pattern (i.e., whether the relative ranking of CBGs is preserved across seeds, independent of point-estimate magnitude).

Notably, cross-CBG rank-stability is higher in 2020-2021 than in 2018-2019 for store area, chain loyalty, POI count, and distance, even though the same-period model fit (Table 1 in the main manuscript) is lower in those years. This is not contradictory: fit measures how well the model matches observed reality, whereas

Table S17. Stratified sample balance for the PSO estimator-stability check (300 CBGs).

Borough	Pool %	Sample %
Bronx	18.5%	18.7%
Brooklyn	33.1%	33.3%
Manhattan	15.7%	15.7%
Queens	28.3%	28.0%
Staten Island	4.4%	4.3%

Note: The sample is also balanced by household-income quintile (each quintile within 0.3 percentage points of its 20% target share).

Table S18. Median per-CBG coefficient of variation across ten seeds, by parameter and year.

Year	Store area	Chain loyalty	POI count	POI diversity	Demographic similarity	Distance
2018	0.476	0.478	0.529	0.556	0.507	0.271
2019	0.484	0.483	0.532	0.539	0.517	0.264
2020	0.374	0.315	0.507	0.506	0.510	0.347
2021	0.349	0.312	0.490	0.508	0.518	0.451

this rank-correlation measures how consistently the optimizer converges to the same relative ordering of CBGs regardless of reality. A sharper, more concentrated pandemic-era visit distribution (fewer viable store alternatives per CBG) can produce a more peaked optimization landscape that is easier for PSO to converge on repeatably, independent of whether that landscape fits the data well.

Finally, Table S20 compares the median CBG-level fit in the 300-CBG stability subsample (averaged across the ten seeds) against the original full-sample fit statistics reported in the main manuscript, confirming that the subsample is broadly representative.

9 SAMPLING-BIAS SENSITIVITY ANALYSIS

Considering the potential sampling bias in mobile-location datasets, we assessed whether the main parameter-change results were sensitive to CBG-level sampling bias in the SafeGraph panel. To do so, we repeated the parameter-change analysis after excluding the CBGs with the highest absolute sampling bias values. Specifically, we compared the full analytical sample with two restricted samples: one excluding the top 10% of CBGs with the highest absolute sampling bias and another excluding the top 20% of CBGs with the highest absolute sampling bias.

Table S21 reports the results for the 2019-2020 comparison. The main pandemic-shock results remain highly stable across all three samples.

Table S22 reports the results for the 2020-2021 comparison. The direction of the larger changes remains stable. Sign changes only occur for chain loyalty and demographic similarity, both of which are very small and close to zero in this comparison.

Table S19. Cross-CBG pairwise Pearson correlation between the ten seeds, by parameter and year.

Year	Parameter	Mean pairwise r	Min pairwise r
2018	Store area	0.494	0.370
2018	Chain loyalty	0.410	0.320
2018	POI count	0.378	0.230
2018	POI diversity	0.164	0.084
2018	Demographic similarity	0.110	-0.020
2018	Distance	0.310	0.169
2019	Store area	0.488	0.397
2019	Chain loyalty	0.396	0.294
2019	POI count	0.355	0.255
2019	POI diversity	0.165	0.063
2019	Demographic similarity	0.085	-0.072
2019	Distance	0.331	0.159
2020	Store area	0.607	0.543
2020	Chain loyalty	0.584	0.467
2020	POI count	0.597	0.517
2020	POI diversity	0.287	0.183
2020	Demographic similarity	0.031	-0.102
2020	Distance	0.587	0.474
2021	Store area	0.657	0.569
2021	Chain loyalty	0.496	0.364
2021	POI count	0.710	0.636
2021	POI diversity	0.334	0.247
2021	Demographic similarity	0.107	-0.008
2021	Distance	0.638	0.566

Table S20. CBG-level model fit: stability subsample versus original full sample.

Year	Stability subsample median fit	Full-sample mean fit	Full-sample median fit
2018	0.723	0.686	0.744
2019	0.744	0.694	0.748
2020	0.171	0.232	0.170
2021	0.150	0.165	0.142

Table S21. Sampling-bias sensitivity of calibrated parameter changes from 2019 to 2020.

Parameter	Full sample	Exclude top 10% abs-bias	Exclude top 20% abs-bias
Chain loyalty	+43.46%	+44.19%	+44.25%
Demographic similarity	+2.50%	+2.68%	+3.62%
CBG-store distance	-22.53%	-22.26%	-22.37%
POI count	+27.93%	+28.12%	+28.29%
POI diversity	+12.20%	+11.93%	+12.63%
Store area	+78.58%	+77.71%	+78.08%

Table S23 reports the 2019-2021 comparison. The non-reversion results remain stable after excluding the most sampling-biased CBGs.

Table S22. Sampling-bias sensitivity of calibrated parameter changes from 2020 to 2021.

Parameter	Full sample	Exclude top 10% abs-bias	Exclude top 20% abs-bias
Chain loyalty	+0.26%	-0.10%	-0.20%
Demographic similarity	-0.32%	+0.19%	+0.07%
CBG-store distance	-15.97%	-15.80%	-15.06%
POI count	+13.15%	+13.08%	+12.17%
POI diversity	+3.23%	+3.11%	+2.31%
Store area	+1.34%	+1.48%	+1.53%

Table S23. Sampling-bias sensitivity of calibrated parameter changes from 2019 to 2021.

Parameter	Full sample	Exclude top 10% abs-bias	Exclude top 20% abs-bias
Chain loyalty	+43.84%	+44.05%	+43.96%
Demographic similarity	+2.18%	+2.87%	+3.69%
CBG-store distance	-34.90%	-34.55%	-34.07%
POI count	+44.75%	+44.88%	+43.90%
POI diversity	+15.82%	+15.41%	+15.23%
Store area	+80.97%	+80.33%	+80.81%

Overall, the sampling-bias sensitivity analysis suggests that the main 2019-2020 pandemic-shock findings and the 2019-2021 non-reversion patterns are not driven by the most over- or under-represented CBGs in the mobility panel.

10 SPATIAL AUTOCORRELATION DIAGNOSTICS

Since the analysis is conducted at the CBG level and the socioeconomic clusters exhibit spatial structure, we examined spatial autocorrelation in CBG-level parameter changes. Specifically, we computed global Moran's I for changes in each calibrated parameter across 2018-2019, 2019-2020, and 2020-2021. Because Queen contiguity produced a small number of isolated CBGs, we used five-nearest-neighbor spatial weights. Table S24 reports the global Moran's I values and corresponding permutation-based p-values.

Table S24. Global Moran's I for CBG-level calibrated parameter changes.

Parameter	2018-2019	2019-2020	2020-2021
Store area	0.009 ($p = 0.125$)	0.157 ($p = 0.001$)	0.104 ($p = 0.001$)
Chain loyalty	-0.011 ($p = 0.090$)	0.136 ($p = 0.001$)	0.063 ($p = 0.001$)
POI count	0.018 ($p = 0.014$)	0.101 ($p = 0.001$)	0.046 ($p = 0.001$)
POI diversity	0.000 ($p = 0.493$)	0.073 ($p = 0.001$)	0.037 ($p = 0.001$)
Demographic similarity	-0.003 ($p = 0.368$)	0.006 ($p = 0.205$)	0.002 ($p = 0.382$)
CBG-store distance	0.006 ($p = 0.226$)	0.105 ($p = 0.001$)	0.093 ($p = 0.001$)

Note: Spatial weights are defined using five-nearest-neighbor relationships among CBGs. P-values are obtained from permutation tests.

The pre-pandemic 2018-2019 changes exhibit little evidence of spatial autocorrelation. In contrast, several pandemic-era changes from 2019 to 2020 and from 2020 to 2021 show statistically significant but modest

spatial autocorrelation. For the 2019-2020 changes, the global Moran's I values are 0.157 for store area, 0.136 for chain loyalty, 0.101 for POI count, 0.073 for POI diversity, 0.006 for demographic similarity, and 0.105 for CBG-store distance. For the 2020-2021 changes, the corresponding values are 0.104 for store area, 0.063 for chain loyalty, 0.046 for POI count, 0.037 for POI diversity, 0.002 for demographic similarity, and 0.093 for CBG-store distance.

These diagnostics suggest that pandemic-era shifts in revealed shopping-location patterns had a spatial component. However, the magnitudes of the global Moran's I values indicate that the main findings are not solely driven by strong spatial clustering. Local indicators of spatial association (LISA) could be used in future work to study local neighborhood spillovers and spatially concentrated changes in revealed store-selection patterns.

11 ABBREVIATIONS USED IN THIS PAPER

CBG: Census Block Group

CDF: Cumulative Distribution Function

DID: Difference-in-Differences

GeoDa: Geographical and Spatial Data Analysis Software

GLM: Generalized Linear Model

GWR: Geographically Weighted Regression

MCI: Multiplicative Competitive Interaction

NAICS: North American Industry Classification System

NYC: New York City

OLS: Ordinary Least Squares

POI: Point of Interest

PSO: Particle Swarm Optimization

SA: Simulated Annealing

12 DATA AND CODE AVAILABILITY

The code and pre-processed data used for the analyses in this study are publicly available in the project repository for complete replication and/or further future analysis purposes. The repository is available at: https://github.com/mohsen-bahrami/urban-shopping-mobility-covid-impacts
